# Supplementary material for: PriMAT: Robust multi-animal tracking of primates in the wild
Source: PLoS One. 2026 Apr 30;21(4):e0347669. doi: 10.1371/journal.pone.0347669 (PMC13132186; doi:10.1371/journal.pone.0347669)
Supplement: S1 Appendix — Transfer to other nonhuman primate species and settings, additional technical information on the tracking model and hyperparameter tuning. (PDF) [file pone.0347669.s001.pdf]

Supplementary material

Transfer to other nonhuman primate species and settings

Qualitative examples show that our model is able to detect most nonhuman primates in those videos without further training (Fig. S1 A). However, there are some clear limitations. The low-resolution videos of PanAf500 are very different to the recordings of lemurs and macaques and some individuals are not detected. When individuals get close to the camera, sometimes several detections within the same individual are made. And lastly, some objects that were not present in the training data are sometimes detected as primates (Fig. S1 B). We selected and annotated 100 frames for each of the three newly introduced settings. We excluded videos from which we had taken our qualitative examples, such that the models would not overfit to the specific video. Starting from our model trained on lemurs and macaques, we finetuned each model for 50 epochs with the respective annotated frames. Afterwards, the models were able to avoid the previously described error cases (Fig. S1 C).

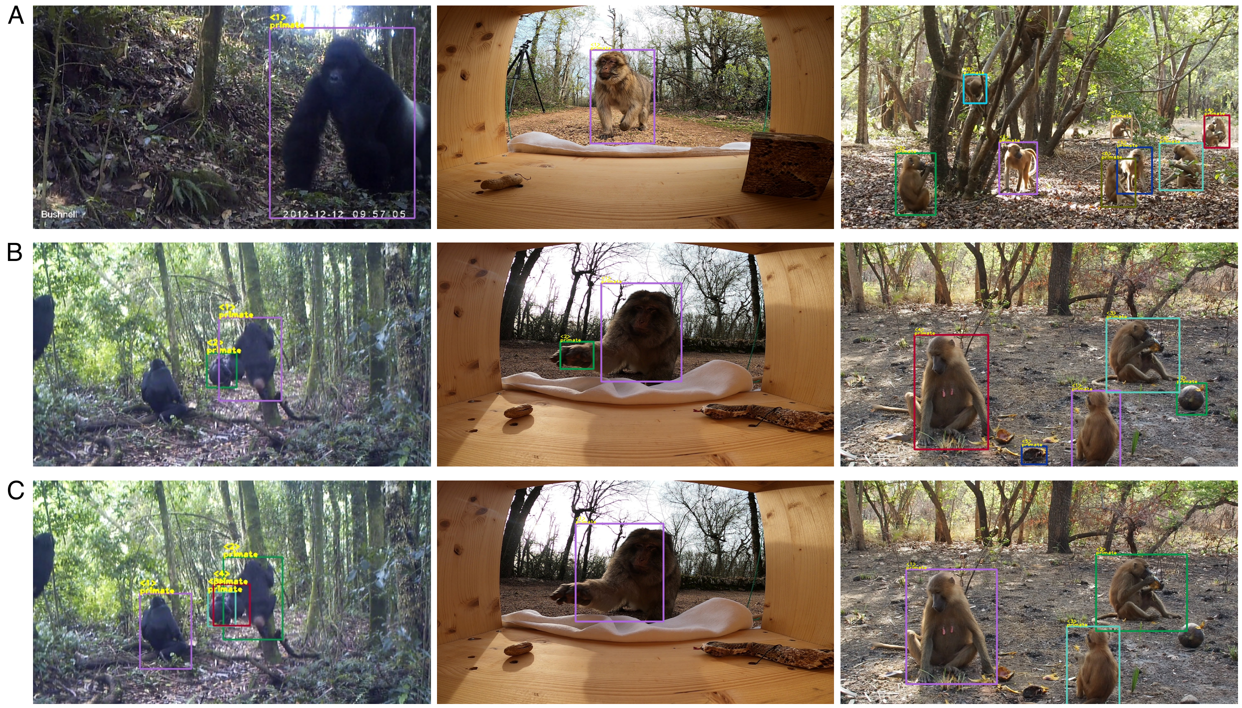

Figure S1: Qualitative examples for transfer to other species. A) Zero-shot performance without training on the target dataset. B) Problems with zero-shot application, false negatives, false positives, additional objects detected. C) Improved model after finetuning on 100 annotated frames from the target dataset.

Tracking model

The backbone processes the input images $I \in [0, 255]^{W_0 \times H_0 \times 3}$ into feature maps $F \in \mathbb{R}^{W \times H \times C}$, where $W = W_0/4$, $H = H_0/4$ (in our case $W \times H = 272 \times 152$ and $C = 512$). We use HRNet [1] as a backbone. Four independent heads process the resulting feature map, each with the same width and height, but with a different number of channels and a different loss, depending on their task. The four heads are:

- Center heatmap (output dimensionality $W \times H \times cl$): This head predicts for each pixel how likely it is to be the center of an object. We adapted the architecture to be a multi-class model by having one center heatmap per class. cl is given by the number of different object classes that should be tracked; in the lemur case $cl = 2$, for tracking lemurs and feeding boxes and $cl = 1$ for the macaque model, as only macaques are being tracked.

- Bounding box size ($W \times H \times 2$): This head predicts for each pixel the width and height of a hypothetical bounding box if there is a center point at this pixel. The two channels correspond to the width and height of the respective bounding box.

- Bounding box offset ($W \times H \times 2$): The feature map F has a resolution four times smaller than the input image size. Therefore, each pixel on the feature map corresponds to 4×4 pixels in the original image. This head predicts a small offset for each predicted bounding box to correct for the effect of down-sampling.

- Re-identification (ReID) features ($W \times H \times 128$): The ReID head generates a numeric representation as a vector for each detected object. This vector encodes the individual's appearance and is later used as additional information for the association of individuals over time.

For more information about the heads and loss functions, please refer to the detailed description in FairMOT [2].

For the association of detections across frames and formation of tracks, the model identifies objects from the local maxima in the center heatmap of the first frame, creating individual tracks. In subsequent frames, it outputs new detections and ReID features, comparing them to existing tracks for similarity. When they match, the detections are added to the corresponding tracks, while unmatched detections are considered potential candidates for new tracks.

The similarity between existing tracks and new detections is assessed using two criteria, appearance and location. Appearance similarity is calculated via cosine similarity of the ReID features, whereas intersection-over-union (IoU) is the measure for similarity in location. Additionally, motion information is modeled using a Kalman filter which predicts the expected location of each bounding box based on velocity in the past frames. The result is a distance matrix between the set of existing tracks (i.e. past detections) and the set of detections in the current frame.

While FairMOT followed a more complicated procedure that involved a sequential application of the two distance measures, we simplified the process as earlier experiments showed that it performed at least equally well. In our model, the final distance matrix is calculated as a linear

combination between the IoU distance matrix and the distance matrix obtained from the appearance features. We found a weight of 0.8 for the IoU distance and 0.2 for the appearance feature distance to work well in our applications. The optimal assignment of new detections to existing tracks is obtained using Hungarian matching [3]. Unmatched tracks are retained for 90 frames to facilitate re-detection after occlusion periods (details see Table S1).

Hyperparameters

The motion patterns and appearance of monkeys and lemurs differ from those of humans. Therefore, PriMAT contains elements that are specifically tailored to videos of primates in the wild. We evaluated how they help to improve performance. We tested hyperparameters to show their influence on model performance (see Table S1).

- Association method: Comparing regular association via Kalman filter and our adaptation for fast motion and jumps, which additionally compares each new detection to the last visible position of the track, if regular association does not find a match.
- Confidence threshold: How confident should the model (i.e. the center heatmap) be to propose a detection at a certain location?
- Detection threshold: How confident should the model be with a detection to start a new track if it was not matched to any existing track?
- Association threshold: We combined IoU and cosine similarity by a linear combination of both matrices. The Hungarian matching returns the association with the highest similarity values between existing tracks and detections. If the best match lies below this association threshold, we leave them unmatched.
- When associating new detections with existing tracks, we calculate a similarity measure from IoU (location) and cosine similarity of the ReID features (appearance). The proportion IoU parameter determines how much importance is given to the location. The importance of appearance is automatically one minus the value for IoU.

Table S1: Performance comparison on 12 validation video sequences. (a) An additional association step to prevent identity switches after jumps or rapid motion improved performance for lemurs, but not for macaques. (b) For pedestrian tracking, a confidence threshold of 0.4 is recommended, but a lower detection threshold improved results for both species. (c) The detection threshold determines whether an unmatched detection can start a new track. (d) The association threshold specifies the minimum similarity required to match a detection to an existing track. (e) The proportion IoU parameter controls the relative weighting of location and appearance during matching. (f) The track buffer defines how long unmatched tracks are retained to allow recovery after occlusion; in our 30 fps videos, any value above 10 frames performed equally well.

	**Lemurs**			**Macaques**		
	**HOTA**	**MOTA**	**IDF1**	**HOTA**	**MOTA**	**IDF1**
**a) Association**						
Regular	68.2	78.1	84.9	**66.6**	**75.5**	**83.7**
Fast motion	**70.3**	**81.5**	**88.1**	64.5	74.0	80.6
**b) Confidence threshold**						
0.01	**70.3**	81.5	**88.1**	64.1	70.7	78.3
0.02	69.8	**81.8**	87.2	65.8	73.8	82.0
0.04	69.4	81.2	86.6	**66.6**	75.5	**83.7**
0.1	69.0	81.2	86.7	65.7	**76.7**	82.2
0.2	68.8	80.0	86.7	65.6	75.8	83.0
0.4	66.9	78.6	84.4	63.0	72.9	78.8
**c) Detection threshold**						
0.4	**70.3**	81.4	88.0	66.1	74.5	82.7
0.5	**70.3**	**81.5**	**88.1**	**66.6**	75.5	**83.7**
0.6	70.2	81.2	87.9	66.5	**76.3**	83.6
**d) Association threshold**						
0.7	**70.3**	**81.5**	**88.1**	**66.6**	**75.5**	**83.7**
0.8	69.5	80.1	86.8	65.4	74.0	82.0
0.9	68.8	78.1	86.4	63.5	72.9	78.9
**e) Proportion IoU**						
0	68.3	77.8	85.3	61.5	72.7	75.6
0.1	69.1	79.3	86.8	63.7	73.5	79.0
0.2	69.3	80.0	86.8	65.0	74.6	81.1
0.3	69.6	81.1	87.1	64.9	75.5	80.5
0.4	69.6	81.0	87.3	**66.6**	75.5	**83.7**
0.5	70.2	81.4	87.8	65.3	75.9	81.0
0.6	**70.3**	**81.5**	**88.1**	65.9	76.6	82.5
0.7	70.2	81.4	88.0	65.9	77.4	82.6
0.8	70.0	81.0	87.8	65.2	**77.5**	80.7
0.9	69.9	80.8	87.6	65.1	77.4	80.6
1	69.9	80.7	87.7	64.9	**77.5**	80.2
**f) Track buffer**						
0	68.6	78.9	85.7	63.3	76.5	75.6
1	68.6	78.9	85.7	63.3	76.5	75.6
5	69.5	79.8	87.0	65.4	**76.9**	80.2
10	70.2	81.2	87.9	66.5	76.8	83.6
30	**70.3**	**81.5**	**88.1**	66.5	76.1	83.5
90	**70.3**	**81.5**	**88.1**	**66.6**	75.5	**83.7**
180	**70.3**	**81.5**	**88.1**	**66.6**	75.5	**83.7**

References

[1] Sun K, Xiao B, Liu D, Wang J. Deep high-resolution representation learning for human pose estimation. In: Proceedings of the IEEE/CVF conference on computer vision and pattern recognition; 2019. p. 5693-703.

[2] Zhang Y, Wang C, Wang X, Zeng W, Liu W. Fairmot: On the fairness of detection and re-identification in multiple object tracking. International Journal of Computer Vision. 2021;129:3069-87.

[3] Kuhn HW. The Hungarian method for the assignment problem. Naval research logistics quarterly. 1955;2(1-2):83-97.
