# Supplementary material for: PriMAT: Robust multi-animal tracking of primates in the wild
Source: PLoS One. 2026 Apr 30;21(4):e0347669. doi: 10.1371/journal.pone.0347669 (PMC13132186; doi:10.1371/journal.pone.0347669)
Supplement: Attachment — Submitted filename: Rebuttal_PriMAT.pdf [file pone.0347669.s003.pdf]

Rebuttal for “PriMAT: Robust multi-animal tracking for primates in the wild”

We thank the reviewers and editors for their constructive comments and updated the manuscript. New sections in the manuscript based on comments from the reviewers / editors are highlighted in red and referenced in the rebuttal using line numbers in the manuscript.

Reviewer 1

> *I have recommended minor revisions. You just need to further separate the introduction from the methods section, as some introductory material is in the methods section. You are also missing some important citations, which I have linked.*

Thank you for the positive feedback and the annotations in the document. We added all recommended citations and moved parts of the methods section to the introduction (L57-67) to improve readability. We additionally extended the discussion of future directions, incorporating some of the recommended recently released benchmarks. The revised paragraph now reads as follows (L530-533):

> The recent datasets PanAf500 (1), BaboonLand (2) or ChimpAct (3) contain activity classes, such as sitting, walking, climbing up or down and would be a suitable benchmark. They could additionally serve as additional pretraining material, such as MacaquePose (4) in our experiments.

Reviewer 2

> *This manuscript could be strengthened by clarifying contribution of this work.*

We clarified our contributions in the introduction (L68-74) as follows.

> Our contributions are four-fold: 1) We propose PriMAT, a multi-animal tracking approach that suits the appearance and motion of monkeys and lemurs, and present two use-cases with highly variable backgrounds and lighting conditions. Both datasets can be used as benchmarks for other methods in the future. 2) We show that the model can easily be adapted to other species and settings by labelling a few hundred frames. 3) We show that our bounding box tracking approach outperforms a keypoint tracking model in the wild. 4) We added a branch that can learn classification tasks on top of the tracking results and used it to identify individual lemurs.

Please note that this new revision does not contain any claim about behavior, although it is the motivation of our method (see next point).

> *The authors assert that bounding box-based tracking is faster to annotation than keypoint detection methods, which is certainly plausible as demonstrated in the experiments and results. However, the authors frame their contribution around behavior studies. Keypoint detections, although more time consuming to annotate, usually generates easy behavior categories "for free" from pose analysis,*

> *where bounding boxes do not. I appreciated the authors quantifying the time taken to annotate the videos using their novel method vs key point detection, however their argument for the new method would be strengthened if behavior annotations were added to this analysis as well. I would suggest evaluating the detections on SOTA behavior-identification models, such as YOLO-Behaviour (Chan et al, 2025, MEE) or X3D from BaboonLand.*

We appreciate the comment and debated how feasible it would be to add behavior to this paper. We came to the conclusion that this is beyond the scope of this paper and would like to detail why.

The reviewer suggested using a action classification method such as YOLO-Behavior or X3D on our data. However, the classical action classes such as standing, walking, sitting etc. are not of relevance for our research question and annotating them to showcase here would be merely an artificial task. The biologically meaningful actions for our videos (looking at, successful sliding, successful pushing, unsuccessful sliding of the feeding box etc.) are all spatio-temporal interactions rather than actions, require different models such as dynamic scene graphs (5; 6) and need careful data curation, which is ongoing research and on the agenda for the next year, but would double the volume of this paper.

Another option would be to apply our method to a published action classification dataset (such as BaboonLand, PanAf500 and ChimpAct) and compare the performance. However, these datasets evaluate action classification independently of tracking and detection, generally on ground truth detections. This means, such an experiment would be disconnected from the rest of our method, which addresses multi-animal tracking. Additionally, it shows that tracking is usually done and evaluated separately from action classification. While both together are needed for detecting animal behavior, our paper focuses on the former. YOLO-Behavior contains only non-primate data and is therefore not in scope.

All the mentioned works (mostly published in 2025 and therefore concurrent work) strengthen our argument: Bounding boxes are very suitable intermediate representations and are finding more and more their way into the analysis of animal behavior on videos.

> *I also suggest revising the manuscript with clearer structure, and clarifying exactly what computer vision tasks are being tackled here - there is a confusing mix of detection, tracking, and individual id, which are all quite different challenges, requiring different evaluation methods.*

We concur that the original structure could be confusing. We improved readability of the manuscript through the following changes:

- We changed the order of presenting our results, and first show everything related to tracking, then the comparison between bounding boxes and keypoints, and in the end, individual identification.
- We aligned the structure of the methods chapter with the results chapter. The section titles are now matching as well (except for the "Training details" which are only in the Methods part), making it easier to follow. Each section matches one of the four contributions stated in the introduction.
- We adapted the overview part of Figure 2 to reflect more clearly the different image and video datasets used for the different tasks. This helps to see where we analyzed detection (image datasets in evaluation) or tracking (video datasets in evaluation).

> *Strengths: - novel datasets - evaluate new method on existing SOTA datasets - good figures showing the datasets and the challenges with Kalman filters (Fig 3)*

> *Weaknesses: - Conversational tone of the work is distracting, please revise in more formal writing style - Manuscript is poorly organized, making the arguments difficult to follow - No analysis of behavior models is done, although behavior studies are emphasized as an important motivation*

We thank the reviewer for the feedback. For the structure and the behavior models, see the answers above. The first section in the methods chapter in the previous version was a mix of introduction (more

conversational) and methods. As suggested by Reviewer 1, we moved parts of this to the introduction, making the style of the methods section hopefully more concise.

> *- very small dataset*

The training sets are small (500 labeled images) by design to be representative for a practical use case. We want to showcase that our model yields robust results even with a few hundred labelled frames. For the validation and test sets we have in total 48 videos of lengths 12 seconds annotated frame by frame, yielding in total (approximately) 17k annotated frames. While this is by far not a large dataset, we believe that it is large enough to showcase the usability of our method.

> *confusing mix of computer vision tasks - detection, tracking, behavior, and individual id is muddled, making it challenging to evaluate the appropriateness of the metrics reported*

We hope that the changes in the structure mentioned above, already help with more clarity. Additionally: The (time-saving) advantage that our model trains on single images and can be applied to videos comes at the cost that different datasets are evaluated differently. In general, we are interested in multi-animal tracking (main metric: HOTA) and apply this wherever possible. In two cases, we have to resort to detection (main metric: AP50) as the best proxy for tracking: when evaluating the copy-paste approaches pairwisely on each other (Fig. 6) as the datasets are image-based. And when transferring to other species (Table 2), as the labelling effort to annotate videos frame-by-frame for each of the additional datasets would be disproportional to the value the insights would bring. The updated Figure 2A shows for which tasks we used image datasets and for which tasks we used video datasets for evaluation.

> *- confused about what exactly the authors mean by "individual id" of lemurs - is this truely re-identification of individuals, or just a more robust tracking method? Why is having the collars in view important? Is there a unique marking on the collars? Please clarify this - a figure or photo could help.*

The collars have a unique combination of different shapes and colors, which is used by the researchers in the field to identify the lemurs. This is why it is important to have them in view for identification. We added a clarifying sentence (L284-286). Our approach is a simple closed-set re-identification method; we added a clarifying sentence (L283-284). Thank you also for the suggestion to clarify this with a figure. We added Figure 5, which shows on two examples that on many frames it is difficult for a model (or a human) to identify the individuals. As we already have tracks, we can use the majority vote across all frames to come to a track-wise decision.

References

[1] Brookes O, Mirmehdi M, Stephens C, Angedakin S, Corogenes K, Dowd D, et al. PanAf20K: a large video dataset for wild ape detection and behaviour recognition. International Journal of Computer Vision. 2024:1-17.

[2] Duporge I, Kholiavchenko M, Harel R, Wolf S, Rubenstein DI, Crofoot MC, et al. BaboonLand Dataset: Tracking Primates in the Wild and Automating Behaviour Recognition from Drone Videos: I. Duporge et al. International Journal of Computer Vision. 2025:1-12.

[3] Ma X, Kaufhold S, Su J, Zhu W, Terwilliger J, Meza A, et al. Chimpact: A longitudinal dataset for understanding chimpanzee behaviors. Advances in Neural Information Processing Systems. 2023;36:27501-31.

[4] Labuguen R, Matsumoto J, Negrete SB, Nishimaru H, Nishijo H, Takada M, et al. MacaquePose: a novel "in the wild" macaque monkey pose dataset for markerless motion capture. Frontiers in behavioral neuroscience. 2021;14:581154.

[5] Jiang X, Zheng C, Xu X, Liu B, Zheng W, Zhang H, et al. VrdONE: One-stage Video Visual Relation

Detection. In: Proceedings of the 32nd ACM International Conference on Multimedia; 2024. p. 1437-46.

[6] Wang G, Li Z, Chen Q, Liu Y. OED: Towards One-stage End-to-End Dynamic Scene Graph Generation. In: Proceedings of the IEEE/CVF Conference on Computer Vision and Pattern Recognition; 2024. p. 27938-47.
