# Supplementary material for: PriMAT: Robust multi-animal tracking of primates in the wild
Source: PLoS One. 2026 Apr 30;21(4):e0347669. doi: 10.1371/journal.pone.0347669 (PMC13132186; doi:10.1371/journal.pone.0347669)
Supplement: Attachment — Submitted filename: PriMAT_Rebuttal_final.pdf [file pone.0347669.s004.pdf]

Rebuttal for "PriMAT: Robust multi-animal tracking for primates in the wild"

We thank the reviewers for their positive and constructive comments and have updated the manuscript. New sections in the manuscript based on comments from the reviewers are highlighted in red and referenced in the rebuttal using line numbers from the manuscript.

Reviewer 1

Reviewer 2

> *Primary Concern: The evaluation would be substantially strengthened by a direct comparison against SAM3, the current state of the art for tracking wildlife in videos. This is particularly important given that SAM3 has been evaluated on camera trap video through the SAFARI benchmark,which is closely aligned with the conditions presented in this work. Without this comparison, it is difficult to assess the proposed method's standing relative to the broader field. The authors should either include this comparison or provide a well-reasoned justification for its omission.*

We thank the reviewer for the suggestion and agree that a comparison adds value to the manuscript. We would also like to mention that this experiment was not possible at the time of submission, as SAM3 (1) was released afterwards. We added the additional experiment to Table 1 and added paragraphs in methods (L223-226; L357-363), results (L418-432), and discussion (L542-554). As expected, SAM3, a large-scale foundation model, achieves strong performance on the macaque dataset and outperforms PriMAT when using the best-performing prompt ("monkey"). However, performance is sensitive to prompt selection: alternative prompts such as "primate" and "macaque" result in a lower HOTA score than PriMAT. This can become an issue for groups that do not have a labelled test set for quantifying performance of different prompts and who have to rely on qualitative results. A second potential issue (depending on the hardware availability and amount of data for inference) is the runtime and memory requirements of SAM3. The runtime increases with additional classes (such as lemurs AND feeding boxes) as each class must be processed in a separate pass. In addition, memory usage depends on the number of detected objects in the video. A third issue is that SAM3, as a foundation model, can not be easily retrained in case more annotated data were available. As a user, one has to work with the performance as it currently is. And lastly, as the applications on the lemur dataset show, it is very difficult to adapt SAM3 to non-standard prompts. The lemurs were evaluated without their large tails; however, the model was not able to segment only the lemur bodies (or we have not found the correct prompt yet). To summarise: Research groups that have a large GPU (A100 or better) available, that want to track standard objects (and not too many classes) and that have a small enough dataset to wait for the inference time, should use SAM3, as it shows remarkably good results in these cases. For all other applications, we see value in using PriMAT.

> *Additional Comments: The abstract should explicitly name the four additional species to which the approach was transferred, rather than leaving this implicit.*

We added this information to the abstract.

> *The comparison of annotation effort across techniques (bounding boxes vs. keyframes) is a valuable contribution and is appreciated.*

Thank you for pointing this out!

> *It should be clarified why models are trained on images but evaluated on video, this methodological choice warrants explicit justification.*

We added a clarifying sentence (L57-60) which reads as follows (with previous sentence for context): [Our model] builds on the FairMOT model, which is trained on individual images and thus does not require entire video sequences to be labeled. This greatly reduces annotation effort, as only selected frames need to be annotated instead of complete videos, while the trained model can still be applied to continuous video during inference.

> *The status of the datasets used is unclear. While citations are provided, the dataset repository suggests these may be new to this publication; the authors should explicitly state whether datasets are previously published or newly introduced here.*

Thank you for pointing out the missing contribution here. The datasets are actually newly introduced in this paper, the citations only refer to the studies during which they were recorded. We added the datasets as a contribution made by our paper (L72-74) which reads as follows: 2) We release two annotated video benchmarks for multi-animal tracking, including videos and frame-level labels for redfronted lemurs and Assamese macaques, comprising 24 videos of 12 s duration each.

> *The authors have adequately addressed feedback from the previous review round.*

We appreciate this comment!

References

[1] Carion N, Gustafson L, Hu YT, Debnath S, Hu R, Suris D, et al. Sam 3: Segment anything with concepts. arXiv preprint arXiv:251116719. 2025.
