# Supplementary material for: PriMAT: Robust multi-animal tracking of primates in the wild
Source: PLoS One. 2026 Apr 30;21(4):e0347669. doi: 10.1371/journal.pone.0347669 (PMC13132186; doi:10.1371/journal.pone.0347669)
Supplement: Attachment — Submitted filename: PONE-D-25-48058_reviewed.docx [file pone.0347669.s002.docx]

PLOS ONE

PriMAT: Robust multi-animal tracking of primates in the wild

--Manuscript Draft--

| Manuscript Number: | PONE-D-25-48058 |
| --- | --- |
| Article Type: | Research Article |
| Full Title: | PriMAT: Robust multi-animal tracking of primates in the wild |
| Short Title: | PriMAT: Robust multi-animal tracking of primates in the wild |
| Corresponding Author: | Alexander Ecker  Georg-August-Universität Göttingen: Georg-August-Universitat Gottingen Göttingen, GERMANY |
| Keywords: | multi-object tracking; multi-animal tracking; individual identification; Deep Learning; Computer vision; animal behavior in the wild |
| Abstract: | Detection and tracking of animals is an important first step for automated behavioral studies using videos. Animal tracking is currently done mostly using deep learning frameworks based on keypoints, which show remarkable results in lab settings with fixed cameras, backgrounds, and lighting. However, multi-animal tracking in the wild presents several challenges such as high variability in background and lighting conditions, complex motion, and occlusion. We propose PriMAT, an approach for tracking nonhuman primates in the wild. PriMAT learns to detect and track primates and other objects of interest from labeled videos or single images using bounding boxes instead of keypoints. Using bounding boxes significantly facilitates data annotation and robustness. Our one-stage model is conceptually simple but highly flexible, and we add a classification branch that allows us to train individual identification. To evaluate the performance of our approach, we applied it in two case studies with Assamese macaques (Macaca assamensis) and redfronted lemurs (Eulemur rufifrons) in the wild. We show that with only a few hundred frames labeled with bounding boxes, we can achieve robust tracking results. Combining these results with the classification branch for the lemur videos, the lemur identification model shows an accuracy of 84% in predicting identities. Our approach presents a promising solution for accurately tracking and identifying animals in the wild, offering researchers a tool to study animal behavior in their natural habitats. Our code, models, training images, and evaluation video sequences are publicly available, facilitating their use for animal behavior analyses and future research in this field. |
| Order of Authors: | Richard Vogg |
|  | Matthias Nuske |
|  | Marissa A. Weis |
|  | Timo Lüddecke |
|  | Elif Karakoc |
|  | Zurna Ahmed |
|  | Sofia M. Pereira |
|  | Suchinda Malaivijitnond |
|  | Suthirote Meesawat |
|  | Derek Murphy |
|  | Julia Fischer |
|  | Florentin Wörgötter |
|  | Peter M. Kappeler |
|  | Alexander Gail |
|  | Julia Ostner |
|  |  |

|  | Oliver Schülke |
| --- | --- |
|  | Claudia Fichtel |
|  | Alexander S. Ecker |
| Additional Information: |  |
| Question | Response |
| Financial Disclosure  Enter a financial disclosure statement that describes the sources of funding for the work included in this submission. Review the [submission guidelines](http://journals.plos.org/plosone/s/submission-guidelines#loc-financial-disclosure-statement) for detailed requirements. View published research articles from [PLOS ONE](https://journals.plos.org/plosone/) for specific examples.  This statement is required for submission and will appear in the published article if the submission is accepted. Please make sure it is accurate. | Yes |
| Funded studies  Enter a statement with the following details:   - Initials of the authors who received each award - Grant numbers awarded to each author - The full name of each funder - URL of each funder website - Did the sponsors or funders play any role in the study design, data collection and analysis, decision to publish, or preparation of the manuscript? |  |
| Did you receive funding for this work? |  |
| Please add funding details.  as follow-up to "Financial Disclosure  Enter a financial disclosure statement that describes the sources of funding for the work included in this submission. Review the [submission guidelines](http://journals.plos.org/plosone/s/submission-guidelines#loc-financial-disclosure-statement) for detailed requirements. View published research articles from [PLOS ONE](https://journals.plos.org/plosone/) for specific examples.  This statement is required for submission and will appear in the published article if | This project was funded by the Deutsche Forschungsgemeinschaft (DFG, German Research Foundation) via project number 454648639 – SFB 1528, project number 254142454 – GRK 2070 and project number 502807174 – RTG 2906.  We acknowledge funding by the Leibniz Association through an Audacity Grant from the Leibniz ScienceCampus Primate Cognition (W45/2019 – Strategische Vernetzung). |

| the submission is accepted. Please make sure it is accurate. |  |
| --- | --- |
| Funded studies  Enter a statement with the following details:   - Initials of the authors who received each award - Grant numbers awarded to each author - The full name of each funder - URL of each funder website - Did the sponsors or funders play any role in the study design, data collection and analysis, decision to publish, or preparation of the manuscript? |  |
| Did you receive funding for this work?" |  |
| Please select the country of your main research funder (please select carefully as in some cases this is used in fee calculation).  as follow-up to "Financial Disclosure  Enter a financial disclosure statement that describes the sources of funding for the work included in this submission. Review the [submission guidelines](http://journals.plos.org/plosone/s/submission-guidelines#loc-financial-disclosure-statement) for detailed requirements. View published research articles from [PLOS ONE](https://journals.plos.org/plosone/) for specific examples.  This statement is required for submission and will appear in the published article if the submission is accepted. Please make sure it is accurate. | GERMANY - DE |
| Funded studies  Enter a statement with the following details:   - Initials of the authors who received each award - Grant numbers awarded to each author - The full name of each funder - URL of each funder website - Did the sponsors or funders play any role in the study design, data collection and analysis, decision to publish, or preparation of the manuscript? |  |
|  |  |

| Did you receive funding for this work?" |  |
| --- | --- |
| Competing Interests  Use the instructions below to enter a competing interest statement for this submission. On behalf of all authors, disclose any [competing interests](http://journals.plos.org/plosone/s/competing-interests) that could be perceived to bias this work—acknowledging all financial support and any other relevant financial or non- financial competing interests.  This statement is required for submission and will appear in the published article if the submission is accepted. Please make sure it is accurate and that any funding sources listed in your Funding Information later in the submission form are also declared in your Financial Disclosure statement.  View published research articles from  [PLOS ONE](http://journals.plos.org/plosone/) for specific examples. | The authors have declared that no competing interests exist. |
| NO authors have competing interests  Enter: The authors have declared that no competing interests exist.  Authors with competing interests  Enter competing interest details beginning with this statement:  I have read the journal's policy and the authors of this manuscript have the following competing interests: [insert competing interests here] |  |
| * typeset |  |
| Ethics Statement  Enter an ethics statement for this submission. This statement is required if the study involved:   - Human participants - Human specimens or tissue | This study adhered to the Guidelines for the Treatment of Animals in Behavioral Research and Teaching and the legal requirements of the countries (Madagascar, Thailand) in which the work was carried out. The Malagasy Ministry of the Environment, the Mention Zoologie et Biodiversité, Animale de l’Université d’Antananarivo and the CNFEREF Morondava approved and authorized this research (036/24/MEDD/SG/DGGE/DAPRNE/SCBE.Re). Data collection in Thailand was authorized by the Department of National Parks, Wildlife and Plant Conservation (DNP) and the National Research Council of Thailand (NRCT) (permit numbers 0401/11121 |

| - Vertebrate animals or cephalopods - Vertebrate embryos or tissues - Field research   Write "N/A" if the submission does not require an ethics statement.  General guidance is provided below. Consult the [submission guidelines](http://journals.plos.org/plosone/s/submission-guidelines#loc-guidelines-for-specific-study-types) for detailed instructions. Make sure that all information entered here is included in the Methods section of the manuscript. | and 0401/13688). |
| --- | --- |

| Format for specific study types  Human Subject Research (involving human participants and/or tissue)   - Give the name of the institutional review board or ethics committee that approved the study - Include the approval number and/or a statement indicating approval of this research - Indicate the form of consent obtained (written/oral) or the reason that consent was not obtained (e.g. the data were analyzed anonymously)   Animal Research (involving vertebrate animals, embryos or tissues)   - Provide the name of the Institutional Animal   Care and Use Committee (IACUC) or other relevant ethics board that reviewed the study protocol, and indicate whether they approved this research or granted a formal waiver of ethical approval   - Include an approval number if one was obtained - If the study involved non-human primates, add additional details about animal welfare and steps taken to ameliorate suffering - If anesthesia, euthanasia, or any kind of animal sacrifice is part of the study, include briefly which substances and/or methods were applied   Field Research  Include the following details if this study involves the collection of plant, animal, or other materials from a natural setting:   - Field permit number - Name of the institution or relevant body that granted permission |  |
| --- | --- |
|  |  |
| Data Availability  Authors are required to make all data underlying the findings described fully available, without restriction, and from the time of publication. PLOS allows rare exceptions to address legal and ethical concerns. See the [PLOS Data Policy](http://journals.plos.org/plosone/s/data-availability) and [FAQ](http://journals.plos.org/plosone/s/data-availability#loc-faqs-for-data-policy) for detailed information. | Yes - all data are fully available without restriction |

| A Data Availability Statement describing where the data can be found is required at submission. Your answers to this question constitute the Data Availability Statement and will be published in the article, if accepted. |  |
| --- | --- |
| Important: Stating ‘data available on request from the author’ is not sufficient. If your data are only available upon request, select ‘No’ for the first question and explain your exceptional situation in the text box. |  |
| Do the authors confirm that all data underlying the findings described in their manuscript are fully available without restriction? |  |
| Describe where the data may be found in full sentences. If you are copying our sample text, replace any instances of XXX with the appropriate details.   - If the data are held or will be held in a public repository, include URLs, accession numbers or DOIs. If this information will only be available after acceptance, indicate this by ticking the box below. For example: All XXX files are available from the XXX database (accession number(s) XXX, XXX.). - If the data are all contained within the manuscript and/or Supporting Information files, enter the following: All relevant data are within the manuscript and its Supporting Information files. - If neither of these applies but you are able to provide details of access elsewhere, with or without limitations, please do so. For example:   Data cannot be shared publicly because of [XXX]. Data are available from the XXX Institutional Data Access / Ethics Committee (contact via XXX) for researchers who meet the criteria for access to confidential data.  The data underlying the results presented in the study are available from (include the name of the third party | All images for model training, all videos for validation and testing, as well as qualitative model output videos are available here: https://data.goettingen-research- online.de/dataverse/sfb1528_z02 (DOI: https://doi.org/10.25625/CMQY0Q)  All codes are available on Github: https://github.com/ecker-lab/PriMAT-tracking |

| and contact information or URL).   - This text is appropriate if the data are owned by a third party and authors do not have permission to share the data.   * typeset |  |
| --- | --- |
| Additional data availability information: |  |

<https://www2.cloud.editorialmanager.com/pone/download.aspx?id=42000459&guid=367cc3f6-46af-4e36-b7f2-32c3c36ae813&scheme=1>Manuscript	[Click here to](https://www2.cloud.editorialmanager.com/pone/download.aspx?id=42000459&guid=367cc3f6-46af-4e36-b7f2-32c3c36ae813&scheme=1)

[access/download;Manuscript;PriMAT Tracking_Monkeys_and_](https://www2.cloud.editorialmanager.com/pone/download.aspx?id=42000459&guid=367cc3f6-46af-4e36-b7f2-32c3c36ae813&scheme=1)

# PriMAT: Robust multi-animal tracking of primates in the wild

Richard Vogg^1,2^, Matthias Nuske^3^, Marissa A. Weis^1^, Timo Lu¨ddecke^1^, Elif Karakoc^2^, Zurna Ahmed^4^, Sofia M. Pereira^5,6^, Suchinda Malaivijitnond^7,8^, Suthirote Meesawat^7^, Derek Murphy^9,10^, Julia Fischer^9,10,11,12^, Florentin W¨org¨otter^3,11,12^, Peter M. Kappeler^2,11,12^, Alexander Gail^4,11,12,14^, Julia Ostner^5,6,11^, Oliver Schu¨lke^5,6,11^, Claudia Fichtel^2,11^, Alexander S. Ecker^1,11,12,15*^

1 Institute of Computer Science and Campus Institute Data Science, University of G¨ottingen, Germany
2 Behavioral Ecology & Sociobiology Unit, German Primate Center, Leibniz Institute for Primate Research, G¨ottingen
3 Department for Computational Neuroscience, Third Physics Institute, University of G¨ottingen
4 Sensorimotor Group, German Primate Center, Leibniz Institute for Primate Research, G¨ottingen
5 Behavioral Ecology Department, University of G¨ottingen
6 Social Evolution in Primates Group, German Primate Center, Leibniz Institute for Primate Research, G¨ottingen
7 National Primate Research Center of Thailand, Chulalongkorn University, Saraburi, Thailand
8 Department of Biology, Faculty of Science, Chulalongkorn University, Bangkok, Thailand
9 Cognitive Ethology Laboratory, German Primate Center, Leibniz Institute for Primate Research, G¨ottingen
10 Department for Primate Cognition, Johann-Friedrich-Blumenbach Institute, University of G¨ottingen
11 Leibniz ScienceCampus, German Primate Center, Leibniz Institute for Primate Research, G¨ottingen
12 Bernstein Center for Computational Neuroscience, University of G¨ottingen
13 Department of Sociobiology/Anthropology, University of G¨ottingen
14 Georg-Elias-Mu¨ller-Institute of Psychology, University of G¨ottingen
15 Max Planck Institute for Dynamics and Self-Organization, G¨ottingen

*^∗^*corresponding author: [ecker@cs.uni-goettingen.de](mailto:ecker@cs.uni-goettingen.de)

* [ecker@cs.uni-goettingen.de](mailto:ecker@cs.uni-goettingen.de)

# Abstract

Detection and tracking of animals is an important first step for automated behavioral studies using videos. Animal tracking is currently done mostly using deep learning frameworks based on keypoints, which show remarkable results in lab settings with fixed cameras, backgrounds, and lighting. However, multi-animal tracking in the wild presents several challenges such as high variability in background and lighting conditions, complex motion, and occlusion. We propose PriMAT, an approach for tracking nonhuman primates in the wild. PriMAT learns to detect and track primates and other objects of interest from labeled videos or single images using bounding boxes instead of keypoints. Using bounding boxes significantly facilitates data annotation and

robustness. Our one-stage model is conceptually simple but highly flexible, and we add a classification branch that allows us to train individual identification. To evaluate the performance of our approach, we applied it in two case studies with Assamese macaques (*Macaca assamensis*) and redfronted lemurs (*Eulemur rufifrons*) in the wild. We show that with only a few hundred frames labeled with bounding boxes, we can achieve robust tracking results. Combining these results with the classification branch for the lemur videos, the lemur identification model shows an accuracy of 84% in predicting identities. Our approach presents a promising solution for accurately tracking and identifying animals in the wild, offering researchers a tool to study animal behavior in their natural habitats. Our code, models, training images, and evaluation video sequences are publicly available^1^, facilitating their use for animal behavior analyses and future research in this field.

# Introduction 1

Automated analysis of actions and social interactions of free-ranging animals is essential	2

for advancing behavioral research, enabling the study of complex behaviors in natural	3

settings. Video recordings are a valuable tool for studying animal behavior, but	4

traditional manual annotation of videos is time-consuming and labor-intensive. In the	5

context of primate behavior research, large-scale annotated datasets have recently	6

enabled the development and training of deep learning based models [1–8]. There are	7

models which show promising results on several computer vision tasks in the wild, such	8

as individual identification [9–12], pose estimation [13, 14], object detection [15] and	9

tracking [16, 17]. However, the vast majority of frameworks used to analyze animal	10

behavior are designed to work in lab settings and rely on pose estimation as an	11

intermediate representation [18, 19]. Three of the most used frameworks for such	12

markerless pose estimation are DeepPoseKit [20], DeepLabCut [21, 22], and SLEAP [23].	13

They allow for multi-animal pose tracking and have user-friendly interfaces that help	14

with annotation and inference. The introduction of these frameworks has contributed to	15

great progress in the area of behavioral analysis.	16

As valuable as these tools are in the lab, their utility for applications of tracking	17

animals in the wild is limited [18, 24]. Recording animals in their natural habitat	18

presents challenges such as occlusion, diverse backgrounds, and varying lighting	19

conditions. Similar appearances between individuals and sudden rapid movements add	20

complexity to the task.	21

We propose a different approach as a step towards analyzing individual actions and	22

social interactions in videos of wild primates. Instead of keypoints as an intermediate	23

representation of the objects, we propose to detect bounding boxes for all animals and	24

relevant objects in the scene (Fig. 1).	25

We see three main reasons why object detection via bounding boxes should be a	26

starting point to consider for many applications. First, currently, there are few	27

successful applications of the above-mentioned keypoint-based frameworks on videos	28

from the wild. DeepWild showed that DeepLabCut can learn from around 2000 labeled	29

frames from videos of animals in the wild; however, the results on unseen videos leave	30

room for improvement [13] and more labels would be necessary to enhance	31

generalization. A study conducted on horse images showed the limited capability of	32

keypoint-based models to generalize to new, unseen horses [24]. A recent survey of	33

keypoint-based methods for animal action classification found that most models do not	34

generalize well to in-the-wild conditions [18]. For humans, pose tracking with variable	35

backgrounds works [25, 26]. The difference here is that diverse, large-scale datasets exist	36

^1^https://github.com/ecker-lab/PriMAT-tracking

**Fig 1.** Bounding box based detection vs. keypoint detection on different datasets. Bounding box based tracking has advantages over keypoint tracking when used as a starting point for automated behavior analysis, such as reduced labeling time, higher tracking robustness for videos from the wild, and universal extensibility to other objects of interest.

and substantial human labor has been put into annotating them [27, 28]. For most	37

animal species, neither large-scale datasets with broad enough coverage exist, nor has an	38

equivalent amount of labor been invested into annotations. Second, using an	39

object-detection framework built on bounding boxes may be preferable because	40

keypoints require significantly more time to annotate than bounding boxes. Wiltshire et	41

al. [13] reported that they needed around two hours to label 18 keypoints for all animals	42

in 25 frames (less than 175 individuals, as videos were chosen to have not more than	43

seven individuals present). We annotated around 200 frames (roughly 1500 bounding	44

boxes) in the same time, which is roughly an order of magnitude more individuals.	45

Third, many questions in animal behavior research, especially in the wild, simply may	46

not require keypoints but instead focus on actions and interactions which can directly	47

be extracted from the image information inside the bounding box.	48

In this paper, we propose PriMAT, a conceptually simple **Pri**mate **M**ulti-**A**nimal	49

**T**racking approach. It builds on the FairMOT model [29], which is trained on individual	50

images and thus does not require entire video sequences to be labeled. Compared to	51

keypoint labeling, it reduces annotation effort, as bounding boxes can be drawn	52

relatively quickly on a few hundred frames, which is enough for transfer learning to	53

other primate species or camera angles. We added a branch that can learn classification	54

tasks, such as individual identification, on top of the tracking results. We present two	55

use cases for our tracking approach by applying it to videos of Assamese macaques and	56

redfronted lemurs in the wild. The models learned to detect and track macaques and	57

lemurs from different camera angles and in videos with highly variable backgrounds and	58

lighting conditions. Additionally, we used the classification branch to identify individual	59

lemurs.	60

# Materials and methods 61

2

2

Identification

Transfer learning is an effective way to reduce annotation effort by starting with a base	62

model pretrained on a large dataset and fine-tuning it with a few hundred annotated	63

frames from the domain of application. We experimented with four pretraining datasets	64

(see below) and annotated 500 frames from videos of lemurs and macaques to	65

demonstrate the applicability of the base model to these species (Fig. 2A). We used	66

tight modal bounding boxes, that is, boxes around the outermost visible pixels of each	67

individual.	68

Tracking

Pretraining

**Fig 2.** (A) Structure of our datasets and experiments. All models were trained with image datasets, while the performance was evaluated primarily on videos. We compared different datasets for pretraining, and applied them to two use cases: videos of redfronted lemurs and Assamese macaques. We compared our bounding box based approach with DeepLabCut [22]. Finally, we demonstrated the use of our additional classification branch for individual identification. (B) Overview of the model architecture: Input images are processed by a Convolutional Neural Network (CNN, in our case HRNet32 [30]) into a feature volume. Different heads learn tracking-related tasks using two-layer CNNs. We omitted the offset head for simplicity. Afterwards, individuals are cut out and processed by the classification branch for individual identification. We used ResNet18 [31] as the CNN for individual recognition.

2

ImageNet [1.2M]

Model 1

MacaquePose MacaqueCopy

2

[10k]

3

Paste(Wild) [20k]

4 (5)

MacaquePose MacaqueCopy Eval [3k]	PasteEval [6k]

MacaqueCopy PasteWildEval

A

Lemur [500]

Macaque [500]

DLC Tracking

Lemur Tracking	Macaque Tracking

Lemur Eval [12]

Lemur Test [12]

Macaque Eval [12]

Macaque Test [12]

LemurID [782]

LemurID

LemurID Eval [30]

Legend Model

Image Dataset [Number images]

Video Dataset [Number videos]

1 Center heatmap

CNN

B

Input

256

2

Bounding box size

H

CNN

W

38

128 Appearance features

CNN

3

Ind1

CNN

Ind2

I

…nd3

36

41

50

62

21

bounding boxes

Feature volume F

Macaque Eval [12]

DeepLabCut

Evaluation

Training

PriMAT

CNN

classification branch

**PriMAT: Multi-animal tracking for primates**	69

PriMAT is based on the FairMOT [29] architecture, a popular multi-object tracking	70

model initially developed for tracking pedestrians. It is structured into three parts (Fig.	71

2B): *(1)* a convolutional backbone used for feature extraction, *(2)* multiple heads that	72

perform different tasks for tracking, and *(3)* a classification branch. More detailed	73

information about backbone, heads and association methodology can be found in S2	74

Appendix.	75

Due to the existing datasets [27, 32] and benchmarks [33–35] for multi-object	76

tracking, FairMOT, like most tracking models, was designed for tracking human	77

pedestrians. However, there are large differences between humans and nonhuman	78

primates, as well as among nonhuman primate species, in appearance, motion patterns,	79

and acceleration. Therefore, we adapted the tracking strategy to make it more suitable	80

for the appearance and motion of monkeys and lemurs as outlined below.	81

Pedestrians in city scenes are generally easily distinguishable from the	82

background [35]. This makes them easier to detect and track than nonhuman primates	83

in their natural habitat, especially when the primates are in non-standard poses or	84

highly occluded. Therefore, we considerably lowered the threshold for potential	85

detections, compared to the original value used for pedestrian tracking. This adjustment	86

was necessary to maintain track continuity, especially in cases where the animals were	87

moving. As low detection thresholds introduce potential false positive detections, we	88

added a threshold to prevent new, unmatched boxes from starting a new track if they	89

have too much overlap with already existing tracks. Additionally, we removed	90

detections less than five pixels away from the image border, as most false positives were	91

found in these areas. The appearance of pedestrians is highly variable between	92

individuals, and therefore, the original FairMOT model uses only learned appearance	93

features when associating existing tracks with new detections. The unmatched tracks	94

and detections are then compared using intersection-over-union (IoU). Nonhuman	95

primates have much less inter-individual variation in appearance; thus, for our use cases,	96

location is a more important predictor than appearance. For this reason, we use a linear	97

combination of appearance and location similarity in one step to calculate association.	98

Nonhuman primates also exhibit greater variability in acceleration and movement	99

patterns compared to pedestrians. Tracking models typically use a Kalman filter to	100

predict the location of objects from frame to frame. The Kalman filter assumes linear	101

motion of objects and predicts the position of an object based on the velocity over the	102

previous frames, which may not be appropriate for tracking nonhuman primates. For	103

instance, when jumping, a lemur moves with high velocity, which then drops close to	104

zero once the animal lands. This kind of movement can cause problems when	105

calculating the association (Fig. 3). To avoid problems with fast motion changes, we	106

added a conditional second stage to the association procedure. If there is no match at	107

the location predicted by the Kalman filter, we additionally check for matches at the	108

location of the last detection.	109

**Data, pretraining and transfer learning**	110

**Pretraining.**	111

The goal of the pretraining is to use already existing annotated datasets that are either	112

general-purpose – covering a wide range of visual categories – or close to the domain of	113

application. We compared ImageNet [36], MacaquePose [3] and two augmented versions	114

of MacaquePose for pretraining. ImageNet is a standard dataset for pretraining,	115

containing over 1.2M images from a variety of object classes. MacaquePose consists of	116

13,083 images of macaques, mostly taken from zoos, and is completely annotated with	117

Predicted bounding boxes

Kalman filter estimates for bounding box centers

**Fig 3.** Problem during a jump: The Kalman filter predicts the location of the lemur track based on past velocity. The detection in the last frame cannot be matched to the lemur track. Top row: Detected bounding boxes and assigned tracks identified by the color of the bounding box. The track of the jumping lemur is lost in the last frame (black) and a new track is instantiated (green). Bottom row: Kalman filter predictions for the expected position of the lemur. The Kalman filter assumes linear motion and predicts a different position when the jump ends (black). The newly instantiated track starts with default parameters for the Kalman filter prediction (green).

instance segmentation masks and keypoints. We constructed bounding boxes from the 118 extreme points of each segmentation mask. Although MacaquePose contains images of 119 animals in outdoor situations, the dataset differs from our forest video domain. To	120

enhance generalization and increase background diversity, we applied copy-paste	121

augmentation to MacaquePose using the labeled instance masks (Fig. 5A). We created	122

two augmented datasets: MacaqueCopyPaste, in which individuals were pasted onto	123

ImageNet backgrounds (Fig. 5A), and MacaqueCopyPasteWild, where backgrounds	124

were taken from the Phu Khieo Wildlife Sanctuary in Thailand. Previous work has	125

shown that copy-paste augmentation improves model performance in instance	126

segmentation [37, 38] and object detection [39, 40], offering an efficient way to generate	127

realistic training images across diverse backgrounds. We split the three datasets	128

MacaquePose, MacaqueCopyPaste, and MacaqueCopyPasteWild into 80% training data	129

and 20% test data and trained one model on each of them. We evaluated each model on	130

the test set of all three datasets using mean Average Precision (mAP), a classical metric	131

for object detection tasks [32, 41].	132

## Assamese Macaques. 133

Data on Assamese macaques were collected as part of a long-term project on the	134

behavioral ecology of wild Assamese macaques in the Phu Khieo Wildlife Sanctuary,	135

North East Thailand [42]. The study groups were all habituated to the presence of	136

human observers for a long-term investigation that started in 2005, and animals did not	137

show any signs of disturbance during focal animal follows or video recording. As a	138

general rule, human observers stay at 3-5m away from the animals at all times. At no	139

time were the macaques fed, trapped, or handled in any other way. Data were collected	140

on approximately 250 individually recognizable subjects of different age-sex classes	141

organized in up to five large multi-male/multi-female social groups ranging in size from	142

40 to 73 individuals (unpublished data). The habitat consists of dense hill evergreen	143

forest, and the monkeys spend the vast majority of their time in the trees, reducing	144

visibility and creating difficult video-recording conditions. The videos were	145

hand-recorded with resolutions of 1920 *×* 1080 using a GoPro Hero 10 camera and	146

3840 *×* 2160 using a Google Pixel 4 camera. More than 300 video snippets have been	147

obtained so far. The videos consist of sequences that are challenging for a tracking	148

model, as they can contain motion blur through camera or animal movement, severe	149

occlusion, and high variability in backgrounds and lighting conditions.	150

We manually annotated the bounding boxes on 500 frames randomly sampled from	151

the macaque videos. To extract meaningful samples, we used the model pretrained on	152

MacaquePose, which yielded an approximate count of individuals in each video. Then,	153

we sampled images from the macaque videos according to the macaque count, i.e. we	154

chose more examples from crowded scenes and fewer from scenes with lone individuals.	155

We used the software VoTT^2^, a user-friendly image annotation tool to label training	156

frames.	157

## Redfronted Lemurs. 158

We recorded videos during a social learning experiment in Kirindy Forest,	159

Madagascar [43]. Elif Karakoc, Richard Vogg, Alexander Ecker, Peter M. Kappeler, and	160

Claudia Fichtel were also involved in the experiment. The study adhered to the	161

ASAB/ABS Guidelines for the Treatment of Animals in Behavioural Research and	162

Teaching, as well as the legal requirements of the country (Madagascar), where the study	163

was carried out. The research protocol was approved by the Malagasy Ministry of the	164

Environment, Water, and Forests (064,22/ MEDD/ SG/ DGGE/ DAPRNE/ SCBE.Re).	165

Cameras were placed before the lemurs came to the experiment (for the exact setup,	166

please see the information below). Red-fronted lemurs participated voluntarily in the	167

experiments and were free to approach the cameras, which they occasionally did, or	168

avoid the cameras. Hence, red-fronted lemurs were not handled during image collection.	169

The experiment involved four groups of redfronted lemurs, with group sizes ranging	170

from five to eight individuals. In each lemur group, we presented four feeding boxes in	171

each experiment and repeated the experiments ten times over the course of three	172

months. The duration of each experiment varied from 10 to 30 minutes. We placed	173

eight GoPro Hero 10 cameras to record the interactions of individuals with the feeding	174

boxes from different angles (Fig. 4A). All cameras recorded videos with a resolution of	175

1920 *×* 1080 and 30 frames per second. Four cameras were placed to record the opening	176

mechanism of each box from a distance of one meter (Fig. 4B). Two cameras mounted	177

on nearby trees were used to capture a top-down view of pairs of boxes (Fig. 4C).	178

Additionally, we placed two cameras on tripods to capture an overview of the	179

experimental set-up from two opposite sides (Fig. 4D). Each video was treated	180

independently by the model, without taking into account input from the other	181

perspectives. We did, however, train one model to work well on all camera perspectives,	182

instead of training three specialized models.	183

We recorded eight videos from different perspectives for each of the 40 experimental	184

sessions. Additionally, we conducted pre-experiment sessions with open boxes so the	185

lemurs could get used to the experimental setup. We used a subset of 50 videos from	186

the pre-experiment sessions to obtain our training set. As the close-up view showed the	187

most variability in lemur appearance, we used 24 videos from this view and 13 videos	188

each from the far view and top views. In each video, we determined the longest segment	189

^2^https://github.com/microsoft/VoTT

B

A

5-7 m

C

D

**Fig 4.** (A) Setup for social learning experiments: Four feeding boxes were placed on the ground and eight cameras were filming from three different perspectives ((B) close,

(C) top, (D) far).

that continuously contained at least one lemur, and randomly extracted 10 frames from	190

each of these segments. The 500 resulting frames formed the training set. In these	191

images, we followed the same procedure as for the macaques and annotated all lemurs	192

by manually drawing the bounding boxes using VoTT. We decided to exclude the lemur	193

tails from the bounding boxes, as they would otherwise occupy a relatively large	194

proportion of the enclosed space and lead to fast changes in bounding box size and	195

location from frame to frame. Additionally, we labeled all feeding boxes in the videos to	196

showcase the multi-class capabilities of our model.	197

## Tracking evaluation. 198

We followed the same evaluation strategy for both species. For each species, we	199

annotated 12 sequences, each one 12 seconds long, frame-by-frame with 30 frames per	200

second, to evaluate the tracking performance with different hyperparameters and	201

settings. For the lemur experiment, we additionally annotated the feeding boxes with	202

bounding boxes. Similarly, we annotated 12 sequences for each species of the same	203

length to form a test set, which can be used for benchmarking against our method.	204

Validation and test videos were taken from experiments that were not used to extract	205

training images. For the macaques, the sequences had varying levels of environmental	206

complexity, and each included some motion or occlusion. For the lemurs, we chose the	207

same number of videos from each camera perspective. For quality control of the	208

annotations used for evaluation, we assessed the consistency between the labels	209

produced by two human annotators on a subset of three videos of the macaque dataset.	210

We matched the annotated bounding boxes using Hungarian matching and calculated	211

the average intersection over union across all boxes. The average IoU score was over 0.8	212

for each of the three videos.	213

We used the video annotation tool CVAT^3^ to label the evaluation and test videos.	214

CVAT offers the advantage of automatically interpolating bounding boxes between	215

frames, reducing the need for manual annotation when consecutive frames remain	216

unchanged. Each primate in every frame was annotated if the annotator was able to	217

recognize it as a primate in the still image. If a primate was temporarily occluded, it	218

was labeled with the same ID after reappearing.	219

^3^https:/[/www.cvat.ai](http://www.cvat.ai/)

We evaluated the performance of our models with several metrics commonly used for	220

multi-object tracking benchmarks. The most important metric is considered to be	221

Higher Order Tracking Accuracy (HOTA) [44], as it gives equal importance to detection	222

and association. Additionally, we report Multi-Object Tracking Accuracy (MOTA) [45]	223

and IDF1 [46], two traditional metrics for easier comparison to other tracking models.	224

**Bounding box and keypoint based detection**	225

In light of the studies mentioned in the introduction suggesting that models for keypoint	226

estimation in the wild struggle with generalization, we performed a small experiment.	227

The goal of this experiment is a fair comparison between methods, helping practitioners	228

determine which approach is more suitable for their setting. For most primate settings,	229

directly applicable models are unavailable, requiring models to learn to generalize to a	230

new domain. This requires data annotation. To ensure fairness, we allocated a fixed	231

annotation time budget and assessed whether reliable performance on unseen videos	232

could be achieved with the resulting annotated data.	233

We compared our lemur model with keypoint-based tracking by training a	234

DeepLabCut model [22] to perform keypoint tracking on our lemur videos (Fig. 7A).	235

We allocated five hours for annotation – the same time required to label 500 frames	236

with bounding boxes – and annotated 33 keypoints per individual in 100 frames. We	237

had annotated 500 frames for the bounding box model by selecting ten frames from	238

each of 50 videos. For the subset, we randomly selected two out of the ten annotated	239

frames from each of the 50 videos, ensuring a comparable background variability.	240

We evaluated the accuracy of keypoint tracking on seven selected keypoints (nose,	241

head, neck, spine, start tail, mid tail, end tail) by annotating additional validation	242

frames from the same 50 videos used for extracting training images. For this, we	243

followed the metric suggested in DeepLabCut, and calculated the root-mean squared	244

error for each detected keypoint to the closest ground truth detection [22]. We used a	245

simple metric to compare detection performance from the bounding box and the	246

keypoint tracking approaches, as we only had bounding box ground truth labeled for	247

complete video sequences. For each ground truth bounding box, we determined whether	248

there was a predicted bounding box with an intersection-over-union (IoU) value of at	249

least 0.5, a standard threshold to decide whether a predicted bounding box should be	250

considered to represent a ground-truth bounding box based on the amount of overlap	251

between them [41]. We then counted the number of predicted bounding boxes that	252

overlapped sufficiently with each ground truth box (i.e. the number of correct	253

detections). Similarly, we counted how often there were at least two ground-truth	254

keypoints within a predicted bounding box. As the keypoints could be close to the box	255

border, we also counted the keypoints that were within the 1*.*5*×* buffered bounding box.	256

The value of 1.5 was chosen as it is a generous extension of the bounding box to allow	257

even imprecisely detected keypoints to count towards a correct detection. For both	258

approaches, we reported the proportion of correct detections out of the total number of	259

ground truth bounding boxes as the recall value for each video.	260

Additionally, we calculated the precision of each model as the proportion of correct	261

detections based on predicted keypoints or bounding boxes, respectively, out of the total	262

number of predictions from each video. We reported the F1 score in both cases,	263

2 *× precision × recall*

*F* 1 =

*.*

*precision* + *recall*

**Transfer to other species**	264

We trained a model jointly with the 500 images from lemur and 500 images from	265

Assamese macaque videos to test its applicability to other settings or other primate	266

species. We tested the model on videos of Barbary macaques (*Macaca sylvanus*) that	267

were recorded as part of a decision-making experiment in Rocamadour, France [47],	268

videos of Guinea baboons (*Papio papio*) ranging near the CRP Simenti Field Station in	269

the Niokolo Koba National Park in Senegal [48], and videos from the PanAf500 dataset,	270

which includes chimpanzees (*Pan troglodytes*) and gorillas (*Gorilla spp.*) [49].	271

Qualitative results from applying the model to unseen videos can be found in S1	272

Appendix. We evaluated the detection performance with a 5-fold cross-validation. As we	273

were operating with annotated images, we reported mean Average Precision (mAP) as a	274

standard detection metric. We additionally reported AP50, a relaxed variant of mAP,	275

where a bounding box counts as correctly predicted if it has an IoU of at least 0.5 with a	276

ground truth bounding box. Models that score high on AP50 but low on mAP are good	277

at finding the presence of an animal, but do not have perfectly precise bounding boxes.	278

**Individual identification**	279

To demonstrate how the tracking approach can be extended, we added a new branch	280

and trained it to identify individual lemurs. All lemurs wore collars with tags of	281

different shapes and colors to identify individuals. To train a model on the individual	282

identification task, we manually selected 350 frames from the close-up videos of one of	283

the groups with six individuals as follows. Using 40 videos from this group, we	284

extracted frames in which the collar of at least one individual was visible. At maximum,	285

we selected 25 frames from a single video to ensure diversity in background and lighting	286

conditions. Again, we used VoTT to annotate the bounding boxes, this time labeled	287

with each lemur’s identity. If any lemurs were present in these frames whose collars were	288

not visible, we annotated them with the label “Unsure”. To evaluate the identification	289

performance on temporal tracks, we selected ten close-up camera videos and extracted	290

three one-minute video sequences from each. As we were interested in assessing	291

identification performance – not tracking – we manually chose sequences in which the	292

lemur tracking model had no ID switches. When the tracking model produces an ID	293

switch, this means that two different lemurs are merged together into one track (e. g.	294

the box switches from individual A to individual B when they pass by or occlude each	295

other). In such cases the identification model cannot classify correctly, as there are two	296

individuals in a single track. We used 30 such one-minute-long videos, containing 92	297

individuals, to evaluate individual identification. We evaluated track-wise results and	298

used accuracy as a performance metric.	299

It is important to note that we were not aiming for frame-wise correct identification,	300

which would have been infeasible, as the lemurs were frequently in positions where	301

identification was impossible. Instead, we used the tracks to arrive at a majority vote	302

on which lemur is visible in each sequence of detections. This required the model to be	303

confident in situations where the lemur is clearly identifiable and not be overconfident	304

in situations where the decision could not be made. The final decision for which	305

individual is predicted for a track was made by exponentially weighting and summing	306

the confidences for each frame in a majority vote.	307

We trained the classification branch for individual identification using a	308

ResNet18 [31] model pretrained on the ImageNet [36] dataset. We fine-tuned the	309

network with the individual lemur images, which we cut out from the training images.	310

As ResNet18 expects square images as input, we used squared bounding boxes to avoid	311

squeezing the rectangular bounding boxes that we had previously annotated into	312

squared shapes and introducing biases to the training set. We achieved the squared	313

bounding boxes by increasing the size of the shorter sides of the previously annotated	314

bounding boxes to match the longer sides and then cropped and resized them to	315

224 *×* 224 pixels. To account for the shift from the perfectly annotated bounding boxes	316

used during training to slightly imperfect bounding boxes predicted during inference, we	317

applied random jitter of up to five pixels to the bounding boxes and random zoom	318

increasing the width and height up to 20% during training. These values were chosen	319

based on a randomized search over the hyperparameter space.	320

We trained the model with 315 hand-selected images of six individuals. The images	321

were extracted from videos in situations where at least one individual was identifiable,	322

either by their face or collar. After training, we used the model to acquire more training	323

data, by applying it to four additional recordings of experiments. We then created a	324

second training set by randomly sampling 20 frames per track and selecting images	325

manually, only including those that had no errors and were not near duplicates of other	326

selected images. This process resulted in 782 training images. Additionally, we used a	327

small validation set of 35 hand-selected images for selecting the model via early	328

stopping and evaluated the models on 30 one-minute-long snippets.	329

**Training details**	330

PriMAT was implemented in PyTorch [50] and trained with the Adam optimizer [51].	331

The training of the base model with MacaquePose (and the MacaqueCopyPaste	332

datasets) was performed on four NVIDIA Quadro RTX 5000 GPUs for two days. We	333

started with a learning rate of 5 *×* 10*^−^*^5^ and reduced it by a factor of 10 after 100 epochs,	334

with a batch size of 8. For fine-tuning with 500 images for both lemurs and macaques,	335

training on one GPU took six hours. The initial learning rate was 10*^−^*^5^. Since training	336

was performed on images while evaluation was conducted on videos, we saved	337

intermediate model checkpoints to monitor progress and reduce the risk of overfitting.	338

For the individual identification task, we cropped the images to include only the	339

bounding boxes surrounding lemurs in the dataset and stacked them afterwards,	340

allowing training with a batch size of 128. The model was trained on a single NVIDIA	341

Quadro RTX 5000 GPU, using early stopping with a patience of 10 epochs. Training	342

time with a learning rate of 2 *×* 10*^−^*^5^ was one hour.	343

Additionally, we trained a model with DeepLabCut [22] version 2.3.8 for which we	344

annotated 33 keypoints per individual. We tested different hyperparameters for pcutoff	345

(confidence for a detection) and IoU. Additionally, we compared DLCRNet ms5 (the	346

data-driven individual assembly method which was recommended for improved	347

performance in the multi-animal setting) and ResNet50 as backbones. We chose the	348

best performing model with DLCRNet ms5, pcutoff 0.0 and IoU 0.6. All models were	349

trained for 200,000 training iterations.	350

**Results**	351

With our multi-animal tracking approach, PriMAT, we were able to train models to	352

detect and track individual macaques and lemurs in videos from the wild by only	353

annotating bounding boxes on a few hundred frames. The approach was specifically	354

designed for good performance in primate videos, with solutions for non-standard poses,	355

occlusion, similar appearance, and jumps (see methods). We evaluated the tracking	356

performance of our models with annotated video data from Assamese macaques in	357

Thailand and redfronted lemurs in Madagascar.	358

## Pretraining and transfer learning. 359

We experimented with a simple copy-paste strategy for improved generalization	360

(examples see Fig. 5A). When evaluating in-domain, we noticed that	361

MacaqueCopyPaste and MacaqueCopyPasteWild showed better results (Fig. 5B) than	362

the original MacaquePose [3] dataset, suggesting that these two datasets are “easier”	363

than MacaquePose. Copying and pasting introduces sharper edges and often the	364

animals are more salient against the background, compared to MacaquePose. We also	365

evaluated how well the models performed across domains. While the models trained	366

with MacaqueCopyPaste performed well on all three datasets, MacaquePose showed	367

weaker results in the other domains. MacaqueCopyPasteWild performed well on	368

MacaquePose but poorly on MacaqueCopyPaste (Fig. 5C). Both copy-paste approaches	369

outperformed the models trained on MacaquePose (Fig. 5D). Since MacaqueCopyPaste	370

offered greater background variability and matched or exceeded the others in	371

performance, we used it for all subsequent experiments.	372

**Fig 5. Pretraining with MacaqueCopyPaste.** (A) Examples from

MacaquePose [3] (top), MacaqueCopyPaste with ImageNet [36] backgrounds (middle), and MacaqueCopyPasteWild with backgrounds from Thailand (bottom). (B) Models trained on the copy-paste approaches yield higher results when tested *in-domain*, i. .e. on their own test sets. This can probably be attributed to sharper edges and more saliency against the background. (C) Results of testing on different test sets relative to the in-domain performance from panel B. MacaqueCopyPaste detects macaques well on all three datasets. MacaquePose did not generalize well to other datasets. (D) Both copy-paste strategies outperform MacaquePose on the macaque validation videos. Error bars: Standard error of the mean from training three models with different random seeds.

We further investigated the ability of the base model pretrained on	373

MacaqueCopyPaste to do transfer learning. We fine-tuned the base model with the 500 374 annotated images of lemurs and macaques and did an extensive hyperparameter search 375 for each species (S3 Appendix).	376

Pretraining with ImageNet (70.3 HOTA) was more beneficial than	377

MacaqueCopyPaste (65.7 HOTA) for the lemurs (Table 1). The domain shift from the	378

images of MacaquePose to the lemur videos is large, not only because the species look	379

different, but the lemur experiments were filmed from different camera angles. For the	380

macaques however, pretraining with MacaqueCopyPaste (66.6 HOTA) was slightly more	381

beneficial than with ImageNet (64.6 HOTA).	382

As we did a lot of hyperparameter tuning on the validation videos, we additionally	383

provide 12 test sequences for each species. These can be used for benchmarking other	384

methods against ours in the future. For the lemurs, the backgrounds and lighting	385

conditions vary from experiment to experiment, however, the general setup is similar.	386

The HOTA score on the test set (68.2) is similar to the validation set (70.3). For the	387

macaques, the videos filmed from handheld cameras show higher variability. While the	388

best performing model achieved 66.6 HOTA on the validation set, the score on the test	389

set is 61.6 (see Table 1).	390

Additionally, we ran experiments on how the size of the training set influences	391

performance. For this, we trained models with subsets of the 500 frames to show their	392

usability even with fewer annotated frames (Fig. 6). For the lemurs, performance with	393

100 frames varies between 58.3 and 60.6 HOTA. The performance of the five macaque	394

models trained with 100 frames ranges between 58.1 and 60.2 HOTA. This shows that	395

the training of the bounding box models converges to stable solutions, which do not	396

show a large variance between different subsets.	397

## Table 1. Performance of Lemur and Macaque tracking models.

|  | **Lemurs** | | | **Macaques** | | |
| --- | --- | --- | --- | --- | --- | --- |
|  | **HOTA** | **MOTA** | **IDF1** | **HOTA** | **MOTA** | **IDF1** |
| **Pretraining (Validation)**  ImageNet (IN) MacaqueCopyPaste (MCP) no pretraining | **70.3**  65.7  61.3 | **81.5**  67.8  64.8 | **88.1**  79.3  77.0 | 64.6  **66.6**  59.3 | 75.0  **75.5**  62.3 | **83.7**  **83.7**  75.5 |
| **Pretraining (Test)**  IN / MCP | 68.2 | 74.2 | 83.6 | 61.6 | 69.1 | 79.9 |

Models were pretrained on different large-scale datasets and subsequently finetuned with 500 annotated images of lemurs or macaques. The tracking performance was evaluated on 12 validation video sequences. MacaqueCopyPaste pretraining resulted in improved results in macaque tracking. Pretraining on ImageNet showed better results for lemur tracking. Finally, we report test scores for the best validation setup (pretraining dataset, hyperparameters as detailed in Tab. ) for each species on 12 test video sequences.

## Bounding box and keypoint based detection. 398

We did a comparison between our bounding box based lemur model and a keypoint	399

based detection method, namely DeepLabCut [22]. First, we observed that only 31% of	400

the visible keypoints were detected as many ground-truth skeletons were completely	401

missing or incomplete in the model predictions. Second, among the correctly detected	402

keypoints, the median over the root-mean squared errors of all detections was 49.6	403

pixels. Of course, this value alone is difficult to interpret, as one pixel represents	404

different distances in different views and we observed large variability between videos	405

(Fig. 7B). However, in the lab, DLC models reach median test errors of under 5 pixels	406

with only 100 samples [21] and across different applications [22], which is almost a	407

magnitude less than our model. The relatively large error value from our model was due	408

Lemurs

70

Macaques

70

65	65

HOTA

HOTA

60	60

55

0	100	200	300	400	500

Sample size

55

0	100	200	300	400	500

Sample size

**Fig 6.** Showing tracking results for lemurs and macaques on the test videos when the model is trained with a subset of the training data. We selected five non-overlapping subsets of size 100, and two subsets of size 200. Training with fewer samples will lead to reduced performance, however, we see that even with 100 annotated frames, we get decent tracking results.

to the prediction of multiple incomplete skeletons within an individual and skeletons	409

that were split over two or more individuals.	410

We compared the performance of keypoint models and bounding box models in	411

detecting the presence of a lemur (without punishing for wrong or missing keypoints).	412

Our model outperformed the keypoint based method (Fig. 7C/D). The largest	413

differences were observed in the top view (0.72 vs 0.50) and the close view (0.89 vs	414

0.72), with minor differences in the far view (0.87 vs 0.83). In one video, which	415

contained high-contrast shadows and therefore a greater need for generalization, the	416

keypoint-based method was not able to detect any keypoints in the whole sequence.	417

The performance of our bounding box method decreased on this challenging video	418

compared to other videos, but it still resulted in a final F1 score of 0.62. For a	419

qualitative comparison, we refer to Fig. 8.	420

A model that outputs incomplete skeletons, many missing detections or incomplete	421

tracks is of questionable utility for practical applications. To overcome these types of	422

problem and have a robust and generalizing keypoint model, a lot more data is needed.	423

We performed an additional experiment in which we annotated only seven keypoints	424

per individual and managed to annotate over 200 frames in the allocated five hours.	425

However, this model performed worse than the one with 33 keypoints. This suggests	426

that having a higher number of keypoints with cross-connections is more critical for	427

performance than increasing the number of annotated frames.	428

## Individual identification. 429

We added a classification branch and applied it to identify individual lemurs in the	430

videos. The final model, which combined tracking and identification information, had	431

an identification accuracy of 84%.	432

## Transfer to other species 433

Applying the joint model without any further training showed good results for the	434

Barbary macaques from Rocamadour, even though the setting in these videos appears	435

quite different from the ones in the videos from Thailand and Madagascar. The model	436

was also relatively successful in detecting baboons, chimpanzees, and gorillas. However,	437

A

ground truth

our model

DLC

B	C	D

Median

FN

3776

TP

1712

FP

1724

1.00

0.9

***

training videos

0	100	200	300	400

root mean squared error (rmse)

0.75

0.50

F1 score (keypoints)

0.25

0.00

0.00 0.25 0.50 0.75 1.00

F1 score (bounding boxes)

Camera perspective

close far top

0.8

0.7

F1 Score

0.6

our model	DLC

**Fig 7.** Comparison between our boxes based tracking model and a DeepLabCut (DLC) model [22]. Our model was trained with 500 images from three camera perspectives.

The DLC model was trained using 33 keypoints, resulting in 100 frames. (A) A qualitative example where DLC does not detect most keypoints. (B) DLC missed many keypoints and shows large root-mean squared errors (rmse) for the predicted keypoints. (C, D) Performance difference on the 12 test videos. The evaluation metric was chosen in a favorable manner for DLC, as it counts a detection as positive when there are at least two keypoints correctly detected.

**Fig 8.** Qualitative comparison between PriMAT and DLC. Ground truth bounding boxes are black, PriMAT bounding boxes are yellow. The different colors of the keypoints refer to different detected individuals.

particularly in the PanAf500 dataset, many individuals were not detected. This is likely	438

because the species in this dataset (all great apes) have a very different appearance	439

from lemurs and Assamese macaques, and the camera resolution is much lower than	440

that of our videos. Performance increased after fine-tuning with 100 frames from the	441

target datasets. We observed high AP50 values, which indicated that most individuals	442

were correctly detected. The relatively lower mAP values indicated that the predicted	443

bounding boxes did not fit perfectly around each individual (Table 2). We show	444

qualitative examples in S1 Appendix.	445

## Table 2. Transfer learning results to other species.

| Dataset | mAP | AP50 |
| --- | --- | --- |
| without training |  |  |
| Rocamadour | 0.55 | 0.83 |
| Guinea baboons | 0.22 | 0.56 |
| PanAf500 | 0.21 | 0.38 |
| finetuning (100 frames) |  |  |
| Rocamadour | 0.73 | 0.99 |
| Guinea baboons | 0.41 | 0.77 |
| PanAf500 | 0.47 | 0.81 |

Detection evaluation metrics for models before and after fine-tuning with 100 frames from the target datasets

**Discussion**	446

There are many potential paths towards automated behavior analysis using videos of	447

animals in the wild. Most current methods in the lab use keypoint detection and	448

tracking as a starting point, as those provide a valuable intermediate representation,	449

namely the pose of the animal. However, so far, keypoint detection based models have	450

struggled to generalize to highly diverse backgrounds in the wild. One solution could be	451

to annotate vastly larger and diverse datasets; however, keypoint annotation is a very	452

time-consuming task. Therefore, we proposed an alternative approach based on	453

bounding boxes. We demonstrated that our deep learning based multi-animal tracking	454

model, PriMAT, outperforms previous methods based on keypoints on videos from the	455

wild while requiring significantly less labeling time. Our tracking model is based on the	456

FairMOT architecture [29], which was built to track pedestrians. We addressed	457

primate-specific challenges in the design of our approach, including non-standard poses,	458

occlusion, and fast, non-linear motion.	459

**Key findings and interpretations.** We presented several experiments around	460

pretraining on larger datasets and transferring to new settings and species with small	461

annotated datasets. What do our results suggest for researchers working with primate	462

videos from the wild? First of all, pretraining with a general-domain dataset like	463

ImageNet [36] is a good starting point. However, if annotated data from a similar	464

domain as the intended application is available, such as MacaquePose [3] in our case,	465

performance can be improved even further. We showed that copy-paste approaches can	466

be applied to bring the data closer to the domain of application. However, transferring	467

from macaques to lemurs showed a larger domain shift, and in this case, ImageNet	468

pretraining was the better way to achieve good performance. Until a general-domain	469

dataset with many different monkey species and backgrounds is available, researchers	470

will have to assess each case individually, which pretraining strategies to apply to	471

achieve the best possible performance with a limited labelling budget.	472

Combining tracking with individual identification models can benefit video analysis,	473

as individuals are often not recognizable on single frames. Using tracks, it suffices to	474

have a few frames with high-confidence prediction and propagate the recognized	475

individual to all the other frames in the track.	476

**Limitations.** Bounding boxes can have drawbacks in certain situations. When	477

elongated objects (such as a monkey with its tail outstretched) are tracked, a large	478

portion of the bounding box can be filled with background. We reduced this problem by	479

excluding the tail from the lemur detections. A second downside is that bounding box	480

coordinates themselves are rarely useful for downstream tasks such as action recognition,	481

which is why subsequently used models always need to process the (cut-out) image	482

information. In contrast, keypoint-based models usually operate directly on the	483

keypoint coordinates and can be more lightweight. Lastly, there are cases when	484

keypoint estimation is needed, for example, when analyzing kinematics [52].	485

When applying PriMAT to lemur and macaque videos, we observed that the models	486

had difficulties with tracking individuals when there was a lot of fast, non-linear	487

movement and occlusion, such as in videos of juveniles playing. Our association strategy	488

is rule-based and relies on a Kalman filter for linear movement (see S2 Appendix). While	489

adapting the association logic as done in our model can lead to further improvement,	490

more recent multi-object tracking architectures promise to overcome problems with	491

challenging association by learning associations as part of their architecture instead of	492

following hand-crafted rules [53, 54]. However, such approaches require densely	493

annotated videos for training, which is more costly than the sparse annotations we use.	494

Hence, their feasibility remains to be evaluated. Another limitation of our (and most	495

more recent trackers) is that they process each video frame independently without	496

incorporating temporal information. Integrating temporal context could enhance	497

performance by using motion patterns and improving consistency across frames.	498

In its current form, our classification branch can be used for individual identification	499

only in cases where all identities are known. If there are unknown individuals in the	500

dataset, open-set classification approaches (e.g. via Deep metric learning) [55] are a	501

more promising pathway as they do not require a predefined set of individuals.	502

**Future directions.** While we primarily view the tracking and identification model as	503

a foundation for future action and interaction recognition, it can already offer value in	504

certain use cases, provided a few key prerequisites are met. First, high video quality,	505

including high resolution and a good signal-to-noise ratio, will help to improve	506

performance in both tracking and identification. Second, the degree of background and	507

scene variability influences the amount of training data required; highly different scenes	508

may fall outside the model’s domain and reduce performance. Third, the behavior of	509

the primates will determine how well the tracking works. Excessive group movement,	510

such as individuals crossing paths or frequently switching positions, can hinder the	511

ability of tracking algorithms to reliably distinguish and follow each subject. These	512

prerequisites could, for example, be met in outdoor situations where the camera is	513

filming from within a feeding device, and the question is which individuals are spending	514

how much time at the device, or the camera is filming along a narrow path, and the	515

question is who passes along the path.	516

The flexibility of the model allows the extension of PriMAT to incorporate other	517

tasks such as instance segmentation [56] and ultimately interaction classification [57]	518

between objects when those interactions are detectable in single frames. The	519

classification branch can be used for individual identification but also to classify actions	520

performed by the individuals. In the computer vision community, many models	521

developed for spatio-temporal action recognition, interaction recognition or gaze	522

detection build on bounding boxes as intermediate representations [58–61]. The recent	523

PanAf500 dataset [49] contains nine activity classes, such as sitting, walking, climbing	524

up or down and would be a suitable benchmark. While our primary focus was on two 525 species of nonhuman primates (Assamese macaques and redfronted lemurs), preliminary 526 qualitative experiments suggest that our approach is similarly applicable to videos of 527 other nonhuman primate species (including Barbary macaques, Guinea baboons,	528

chimpanzees and gorillas). We expect the model to generalize to other animal species	529

with little additional annotation effort.	530

# Supporting information 531

## S1 Appendix. Transfer to other nonhuman primate species and settings. 532

Qualitative examples show that our model is able to detect most nonhuman primates in	533

those videos without further training (S1 FigA). However, there are some clear	534

limitations. The low-resolution videos of PanAf500 are very different to the recordings	535

of lemurs and macaques and some individuals are not detected. When individuals get	536

close to the camera, sometimes several detections within the same individual are made.	537

And lastly, some objects that were not present in the training data are sometimes	538

detected as primates (S1 FigB). We selected and annotated 100 frames for each of the	539

three newly introduced settings. We excluded videos from which we had taken our	540

qualitative examples, such that the models would not overfit to the specific video.	541

Starting from our model trained on lemurs and macaques, we finetuned each model for	542

50 epochs with the respective annotated frames. Afterwards, the models were able to	543

avoid the previously described error cases (S1 FigC).	544

**S1 Fig.**	545

A

B

C

546

**Transfer to other species.** Qualitative examples of our model trained jointly on	547

macaques and lemurs, and applied to different species and settings (from left to right:	548

PanAf500 dataset with gorillas and chimpanzees [49], recordings of a decision-making	549

test with Barbary macaques in Rocamadour, France [47], Guinea baboons ranging near	550

the CRP Simenti Field Station in the Niokolo Koba National Park in Senegal [48]). (A)	551

Positive examples without further training on the target dataset. (B) Error cases:	552

missing individuals, additional detection for limbs when approaching the camera,	553

detection of other objects as primates. (C) After retraining with 100 annotated samples	554

from the specific target dataset (selected from different videos), the error cases are	555

reduced substantially.	556

**S2 Appendix Tracking model.** The backbone processes the input images	557

*I ∈* [0*,* 255]*^W^*0*×H*0*×*3 into feature maps *F ∈* R*^W^ ^×H×C^*, where *W* = *W*_0_*/*4, *H* = *H*_0_*/*4 (in	558

our case *W × H* = 272 *×* 152 and *C* = 512). We use HRNet [30] as a backbone. Four	559

independent heads process the resulting feature map, each with the same width and	560

height, but with a different number of channels and a different loss, depending on their	561

task. The four heads are:	562

- Center heatmap (output dimensionality *W × H × cl*): This head predicts for each	563

pixel how likely it is to be the center of an object. We adapted the architecture to	564

be a multi-class model by having one center heatmap per class. *cl* is given by the	565

number of different object classes that should be tracked; in the lemur case *cl* = 2,	566

for tracking lemurs and feeding boxes and *cl* = 1 for the macaque model, as only	567

macaques are being tracked.	568

- Bounding box size (*W × H ×* 2): This head predicts for each pixel the width and	569

height of a hypothetical bounding box if there is a center point at this pixel. The	570

two channels correspond to the width and height of the respective bounding box.	571

- Bounding box offset (*W × H ×* 2): The feature map *F* has a resolution four times	572

smaller than the input image size. Therefore, each pixel on the feature map	573

corresponds to 4 *×* 4 pixels in the original image. This head predicts a small offset	574

for each predicted bounding box to correct for the effect of down-sampling.	575

- Re-identification (ReID) features (*W × H ×* 128): The ReID head generates a	576

numeric representation as a vector for each detected object. This vector encodes	577

the individual’s appearance and is later used as additional information for the	578

association of individuals over time.	579

For more information about the heads and loss functions, please refer to the detailed	580

description in FairMOT [29].	581

For the association of detections across frames and formation of tracks, the model	582

identifies objects from the local maxima in the center heatmap of the first frame,	583

creating individual tracks. In subsequent frames, it outputs new detections and ReID	584

features, comparing them to existing tracks for similarity. When they match, the	585

detections are added to the corresponding tracks, while unmatched detections are	586

considered potential candidates for new tracks.	587

The similarity between existing tracks and new detections is assessed using two	588

criteria, appearance and location. Appearance similarity is calculated via cosine	589

similarity of the ReID features, whereas intersection-over-union (IoU) is the measure for	590

similarity in location. Additionally, motion information is modeled using a Kalman	591

filter which predicts the expected location of each bounding box based on velocity in	592

the past frames. The result is a distance matrix between the set of existing tracks (i.e.	593

past detections) and the set of detections in the current frame.	594

While FairMOT followed a more complicated procedure that involved a sequential	595

application of the two distance measures, we simplified the process as earlier	596

experiments showed that it performed at least equally well. In our model, the final	597

distance matrix is calculated as a linear combination between the IoU distance matrix	598

and the distance matrix obtained from the appearance features. We found a weight of	599

0.8 for the IoU distance and 0.2 for the appearance feature distance to work well in our	600

applications. The optimal assignment of new detections to existing tracks is obtained	601

using Hungarian matching [62]. Unmatched tracks are retained for 90 frames to	602

facilitate re-detection after occlusion periods (details see S1 Table).	603

**S3 Appendix Hyperparameters.** The motion patterns and appearance of monkeys 604 and lemurs differ from those of humans. Therefore, PriMAT contains elements that are 605 specifically tailored to videos of primates in the wild. We evaluated how they help to 606 improve performance. We tested hyperparameters to show their influence on model	607

performance (S1 Table).	608

- Association method: Comparing regular association via Kalman filter and our	609

adaptation for fast motion and jumps, which additionally compares each new	610

detection to the last visible position of the track, if regular association does not	611

find a match.	612

- Confidence threshold: How confident should the model (i.e. the center heatmap)	613

be to propose a detection at a certain location?	614

- Detection threshold: How confident should the model be with a detection to start	615

a new track if it was not matched to any existing track?	616

- Association threshold: We combined IoU and cosine similarity by a linear	617

combination of both matrices. The Hungarian matching returns the association	618

with the highest similarity values between existing tracks and detections. If the	619

best match lies below this association threshold, we leave them unmatched.	620

- When associating new detections with existing tracks, we calculate a similarity	621

measure from IoU (location) and cosine similarity of the ReID features	622

(appearance). The proportion IoU parameter determines how much importance is	623

given to the location. The importance of appearance is automatically one minus	624

the value for IoU.	625

## S1 Table Hyperparameters 626

# Acknowledgements. 627

This project was funded by the Deutsche Forschungsgemeinschaft (DFG, German	628

Research Foundation) via project number 454648639 – SFB 1528, project number	629

254142454 – GRK 2070 and project number 502807174 – RTG 2906. We acknowledge	630

funding by the Leibniz Association through an Audacity Grant from the Leibniz	631

ScienceCampus Primate Cognition (W45/2019 – Strategische Vernetzung). We thank	632

the Department of National Parks, Wildlife, and Plant Conservation (DNP) and the	633

National Research Council of Thailand (NRCT) for research permission. We gratefully	634

acknowledge the computing time granted by the Resource Allocation Board and	635

provided on the supercomputer Emmy/Grete at NHR-Nord@G¨ottingen as part of the	636

NHR infrastructure. The calculations for this research were conducted with computing	637

resources under the project nib00021.	638

# Ethical approval. 639

This study adhered to the Guidelines for the Treatment of Animals in Behavioral	640

Research and Teaching [63] and the legal requirements of the countries (Madagascar,	641

Thailand) in which the work was carried out. The Malagasy Ministry of the	642

Environment, the Mention Zoologie et Biodiversit´e, Animale de l’Universit´e	643

d’Antananarivo and the CNFEREF Morondava approved and authorized this research	644

(036/24/MEDD/SG/DGGE/DAPRNE/SCBE.Re). Data collection in Thailand was	645

authorized by the Department of National Parks, Wildlife and Plant Conservation	646

## Table 3. Hyperparameters

|  | **Lemurs** | | | **Macaques** | | |
| --- | --- | --- | --- | --- | --- | --- |
|  | **HOTA** | **MOTA** | **IDF1** | **HOTA** | **MOTA** | **IDF1** |
| **a) Association** |  |  |  |  |  |  |
| Regular | 68.2 | 78.1 | 84.9 | **66.6** | **75.5** | **83.7** |
| Fast motion | **70.3** | **81.5** | **88.1** | 64.5 | 74.0 | 80.6 |
| **b) Confidence threshold** |  |  |  |  |  |  |
| 0.01 | **70.3** | 81.5 | **88.1** | 64.1 | 70.7 | 78.3 |
| 0.02 | 69.8 | **81.8** | 87.2 | 65.8 | 73.8 | 82.0 |
| 0.04 | 69.4 | 81.2 | 86.6 | **66.6** | 75.5 | **83.7** |
| 0.1 | 69.0 | 81.2 | 86.7 | 65.7 | **76.7** | 82.2 |
| 0.2 | 68.8 | 80.0 | 86.7 | 65.6 | 75.8 | 83.0 |
| 0.4 | 66.9 | 78.6 | 84.4 | 63.0 | 72.9 | 78.8 |
| **c) Detection threshold** |  |  |  |  |  |  |
| 0.4 | **70.3** | 81.4 | 88.0 | 66.1 | 74.5 | 82.7 |
| 0.5 | **70.3** | **81.5** | **88.1** | **66.6** | 75.5 | **83.7** |
| 0.6 | 70.2 | 81.2 | 87.9 | 66.5 | **76.3** | 83.6 |
| **d) Association threshold** |  |  |  |  |  |  |
| 0.7 | **70.3** | **81.5** | **88.1** | **66.6** | **75.5** | **83.7** |
| 0.8 | 69.5 | 80.1 | 86.8 | 65.4 | 74.0 | 82.0 |
| 0.9 | 68.8 | 78.1 | 86.4 | 63.5 | 72.9 | 78.9 |
| **e) Proportion IoU** |  |  |  |  |  |  |
| 0 | 68.3 | 77.8 | 85.3 | 61.5 | 72.7 | 75.6 |
| 0.1 | 69.1 | 79.3 | 86.8 | 63.7 | 73.5 | 79.0 |
| 0.2 | 69.3 | 80.0 | 86.8 | 65.0 | 74.6 | 81.1 |
| 0.3 | 69.6 | 81.1 | 87.1 | 64.9 | 75.5 | 80.5 |
| 0.4 | 69.6 | 81.0 | 87.3 | **66.6** | 75.5 | **83.7** |
| 0.5 | 70.2 | 81.4 | 87.8 | 65.3 | 75.9 | 81.0 |
| 0.6 | **70.3** | **81.5** | **88.1** | 65.9 | 76.6 | 82.5 |
| 0.7 | 70.2 | 81.4 | 88.0 | 65.9 | 77.4 | 82.6 |
| 0.8 | 70.0 | 81.0 | 87.8 | 65.2 | **77.5** | 80.7 |
| 0.9 | 69.9 | 80.8 | 87.6 | 65.1 | 77.4 | 80.6 |
| 1 | 69.9 | 80.7 | 87.7 | 64.9 | **77.5** | 80.2 |
| **f) Track buffer** |  |  |  |  |  |  |
| 0 | 68.6 | 78.9 | 85.7 | 63.3 | 76.5 | 75.6 |
| 1 | 68.6 | 78.9 | 85.7 | 63.3 | 76.5 | 75.6 |
| 5 | 69.5 | 79.8 | 87.0 | 65.4 | **76.9** | 80.2 |
| 10 | 70.2 | 81.2 | 87.9 | 66.5 | 76.8 | 83.6 |
| 30 | **70.3** | **81.5** | **88.1** | 66.5 | 76.1 | 83.5 |
| 90 | **70.3** | **81.5** | **88.1** | **66.6** | 75.5 | **83.7** |
| 180 | **70.3** | **81.5** | **88.1** | **66.6** | 75.5 | **83.7** |

Comparison of tracking performance on the 12 validation video sequences for each, lemurs and macaques. a) An additional association step to prevent identity switches after jumps or fast motion improved performance for lemurs, but not for macaques, where the validation videos did not contain any jumps. b) The confidence threshold for pedestrian tracking is recommended at 0.4. A reduced detection threshold showed improved results for both lemurs and macaques. c) The detection threshold needs to be surpassed for an unmatched detection to start a new track. d) The association threshold is the minimum amount of similarity required between a detection and a track to form a match. e) The proportion IoU parameter manages the relative importance between location and appearance for matching detections and tracks. f) The track buffer determines for how long unmatched tracks are being stored to find a matching detection

e.g. after a period of occlusion. Our videos had a frame rate of 30 and any track buffer over 10 frames performed equally well.

(DNP) and the National Research Council of Thailand (NRCT) (permit numbers	647

0401/11121 and 0401/13688).	648

# References

1. Bala PC, Eisenreich BR, Yoo SBM, Hayden BY, Park HS, Zimmermann J. Automated markerless pose estimation in freely moving macaques with OpenMonkeyStudio. Nature communications. 2020;11(1):4560.
2. Brookes O, Burghardt T. A dataset and application for facial recognition of individual gorillas in zoo environments. arXiv preprint arXiv:201204689. 2020.
3. Labuguen R, Matsumoto J, Negrete SB, Nishimaru H, Nishijo H, Takada M, et al. MacaquePose: a novel “in the wild” macaque monkey pose dataset for markerless motion capture. Frontiers in behavioral neuroscience. 2021;14:581154.
4. Yu H, Xu Y, Zhang J, Zhao W, Guan Z, Tao D. Ap-10k: A benchmark for animal pose estimation in the wild. arXiv preprint arXiv:210812617. 2021.
5. Desai N, Bala P, Richardson R, Raper J, Zimmermann J, Hayden B. OpenApePose, a database of annotated ape photographs for pose estimation. Elife. 2023;12:RP86873.
6. Yang Y, Yang J, Xu Y, Zhang J, Lan L, Tao D. Apt-36k: A large-scale benchmark for animal pose estimation and tracking. Advances in Neural Information Processing Systems. 2022;35:17301-13.
7. Ng XL, Ong KE, Zheng Q, Ni Y, Yeo SY, Liu J. Animal kingdom: A large and diverse dataset for animal behavior understanding. In: Proceedings of the IEEE/CVF Conference on Computer Vision and Pattern Recognition; 2022. p. 19023-34.
8. Ma X, Kaufhold S, Su J, Zhu W, Terwilliger J, Meza A, et al. Chimpact: A longitudinal dataset for understanding chimpanzee behaviors. Advances in Neural Information Processing Systems. 2023;36:27501-31.
9. Schofield D, Nagrani A, Zisserman A, Hayashi M, Matsuzawa T, Biro D, et al. Chimpanzee face recognition from videos in the wild using deep learning. Science advances. 2019;5(9):eaaw0736.
10. Bain M, Nagrani A, Schofield D, Zisserman A. Count, crop and recognise: Fine-grained recognition in the wild. In: Proceedings of the IEEE/CVF International Conference on Computer Vision Workshops; 2019. .
11. Schofield DP, Albery GF, Firth JA, Mielke A, Hayashi M, Matsuzawa T, et al. Automated face recognition using deep neural networks produces robust primate social networks and sociality measures. Methods in Ecology and Evolution. 2023;14(8):1937-51.
12. Paulet J, Molina A, Beltzung B, Suzumura T, Yamamoto S, Sueur C. Deep learning for automatic facial detection and recognition in Japanese macaques: illuminating social networks. Primates. 2024:1-15.
13. Wiltshire C, Lewis-Cheetham J, Komedov´a V, Matsuzawa T, Graham KE, Hobaiter C. DeepWild: Application of the pose estimation tool DeepLabCut for behaviour tracking in wild chimpanzees and bonobos. Journal of Animal Ecology. 2023.
14. Marks M, Jin Q, Sturman O, von Ziegler L, Kollmorgen S, von der Behrens W, et al. Deep-learning-based identification, tracking, pose estimation and behaviour classification of interacting primates and mice in complex environments. Nature machine intelligence. 2022;4(4):331-40.
15. Roy AM, Bhaduri J, Kumar T, Raj K. WilDect-YOLO: An efficient and robust computer vision-based accurate object localization model for automated endangered wildlife detection. Ecological Informatics. 2023;75:101919.
16. Koger B, Deshpande A, Kerby JT, Graving JM, Costelloe BR, Couzin ID. Quantifying the movement, behaviour and environmental context of group-living animals using drones and computer vision. Journal of Animal Ecology. 2023.
17. Pineda RR, Kubo T, Shimada M, Ikeda K. Deep MAnTra: deep learning-based multi-animal tracking for Japanese macaques. Artificial Life and Robotics. 2023;28(1):127-38.
18. Perez M, Toler-Franklin C. CNN-Based Action Recognition and Pose Estimation for Classifying Animal Behavior from Videos: A Survey. arXiv preprint arXiv:230106187. 2023.
19. Vogg R, Lu¨ddecke T, Henrich J, Dey S, Nuske M, Hassler V, et al. Computer Vision for Primate Behavior Analysis in the Wild. arXiv preprint arXiv:240116424. 2024.
20. Graving JM, Chae D, Naik H, Li L, Koger B, Costelloe BR, et al. DeepPoseKit, a software toolkit for fast and robust animal pose estimation using deep learning. Elife. 2019;8:e47994.
21. Mathis A, Mamidanna P, Cury KM, Abe T, Murthy VN, Mathis MW, et al. DeepLabCut: markerless pose estimation of user-defined body parts with deep learning. Nature neuroscience. 2018;21(9):1281-9.
22. Lauer J, Zhou M, Ye S, Menegas W, Schneider S, Nath T, et al. Multi-animal pose estimation, identification and tracking with DeepLabCut. Nature Methods. 2022;19(4):496-504.
23. Pereira TD, Tabris N, Matsliah A, Turner DM, Li J, Ravindranath S, et al. SLEAP: A deep learning system for multi-animal pose tracking. Nature methods. 2022;19(4):486-95.
24. Mathis A, Biasi T, Schneider S, Yuksekgonul M, Rogers B, Bethge M, et al. Pretraining boosts out-of-domain robustness for pose estimation. In: Proceedings of the IEEE/CVF Winter Conference on Applications of Computer Vision; 2021.

p. 1859-68.

25. Xiao B, Wu H, Wei Y. Simple baselines for human pose estimation and tracking. In: Proceedings of the European conference on computer vision (ECCV); 2018. p. 466-81.
26. Xu Y, Zhang J, Zhang Q, Tao D. Vitpose: Simple vision transformer baselines for human pose estimation. Advances in Neural Information Processing Systems. 2022;35:38571-84.
27. Ionescu C, Papava D, Olaru V, Sminchisescu C. Human3. 6m: Large scale datasets and predictive methods for 3d human sensing in natural environments. IEEE transactions on pattern analysis and machine intelligence. 2013;36(7):1325-39.
28. Andriluka M, Iqbal U, Insafutdinov E, Pishchulin L, Milan A, Gall J, et al. Posetrack: A benchmark for human pose estimation and tracking. In: Proceedings of the IEEE conference on computer vision and pattern recognition; 2018. p. 5167-76.
29. Zhang Y, Wang C, Wang X, Zeng W, Liu W. Fairmot: On the fairness of detection and re-identification in multiple object tracking. International Journal of Computer Vision. 2021;129:3069-87.
30. Sun K, Xiao B, Liu D, Wang J. Deep high-resolution representation learning for human pose estimation. In: Proceedings of the IEEE/CVF conference on computer vision and pattern recognition; 2019. p. 5693-703.
31. He K, Zhang X, Ren S, Sun J. Deep residual learning for image recognition. In: Proceedings of the IEEE conference on computer vision and pattern recognition; 2016. p. 770-8.
32. Shao S, Zhao Z, Li B, Xiao T, Yu G, Zhang X, et al. Crowdhuman: A benchmark for detecting human in a crowd. arXiv preprint arXiv:180500123. 2018.
33. Milan A, Leal-Taix´e L, Reid I, Roth S, Schindler K. MOT16: A benchmark for multi-object tracking. arXiv preprint arXiv:160300831. 2016.
34. Dendorfer P, Rezatofighi H, Milan A, Shi J, Cremers D, Reid I, et al. Mot20: A benchmark for multi object tracking in crowded scenes. arXiv preprint arXiv:200309003. 2020.
35. Sun P, Cao J, Jiang Y, Yuan Z, Bai S, Kitani K, et al. Dancetrack: Multi-object tracking in uniform appearance and diverse motion. In: Proceedings of the IEEE/CVF Conference on Computer Vision and Pattern Recognition; 2022. p. 20993-1002.
36. Deng J, Dong W, Socher R, Li LJ, Li K, Fei-Fei L. Imagenet: A large-scale hierarchical image database. In: 2009 IEEE conference on computer vision and pattern recognition. Ieee; 2009. p. 248-55.
37. Ghiasi G, Cui Y, Srinivas A, Qian R, Lin TY, Cubuk ED, et al. Simple

copy-paste is a strong data augmentation method for instance segmentation. In: Proceedings of the IEEE/CVF conference on computer vision and pattern recognition; 2021. p. 2918-28.

38. Barreto A, Reifenrath L, Vogg R, Sinz F, Mahlein AK. Data augmentation for mask-based leaf segmentation of uav-images as a basis to extract leaf-based phenotyping parameters. KI-Ku¨nstliche Intelligenz. 2023:1-14.
39. Dwibedi D, Misra I, Hebert M. Cut, paste and learn: Surprisingly easy synthesis for instance detection. In: Proceedings of the IEEE international conference on computer vision; 2017. p. 1301-10.
40. Yu X, Li F, Bai P, Liu Y, Chen Y. Copy-paste with self-adaptation: A self-adaptive adjustment method based on copy-paste augmentation. IET Computer Vision. 2023;17(8):936-47.
41. Lin TY, Maire M, Belongie S, Hays J, Perona P, Ramanan D, et al. Microsoft coco: Common objects in context. In: Computer Vision–ECCV 2014: 13th European Conference, Zurich, Switzerland, September 6-12, 2014, Proceedings, Part V 13. Springer; 2014. p. 740-55.
42. Schu¨lke O, Pesek D, Whitman BJ, Ostner J. Ecology of Assamese macaques (Macaca assamensis) at Phu Khieo Wildlife Sanctuary, Thailand. Journal of Wildlife in Thailand. 2011;18(1):1-15.
43. Karakoc E, Vogg R, Marziliano M, Petersdorff-Campen J, Ecker A, Kappeler PM, et al. Foraging competence and scrounging tolerance enhance social relationships in a socially tolerant wild primate. bioRxiv. 2025:2025-06.
44. Luiten J, Osep A, Dendorfer P, Torr P, Geiger A, Leal-Taix´e L, et al. Hota: A higher order metric for evaluating multi-object tracking. International journal of computer vision. 2021;129:548-78.
45. Bernardin K, Stiefelhagen R. Evaluating multiple object tracking performance: the clear mot metrics. EURASIP Journal on Image and Video Processing. 2008;2008:1-10.
46. Ristani E, Solera F, Zou R, Cucchiara R, Tomasi C. Performance measures and a data set for multi-target, multi-camera tracking. In: European conference on computer vision. Springer International Publishing Cham; 2016. p. 17-35.
47. Bosshard TC, Mundry R, Fischer J. Ecological risk-taking across age in Barbary macaques. bioRxiv. 2024:2024-07.
48. Fischer J, Kopp GH, Dal Pesco F, Goffe A, Hammerschmidt K, Kalbitzer U, et al. Charting the neglected West: The social system of Guinea baboons. American Journal of Physical Anthropology. 2017;162:15-31.
49. Brookes O, Mirmehdi M, Stephens C, Angedakin S, Corogenes K, Dowd D, et al. PanAf20K: a large video dataset for wild ape detection and behaviour recognition. International Journal of Computer Vision. 2024:1-17.
50. Paszke A, Gross S, Massa F, Lerer A, Bradbury J, Chanan G, et al. Pytorch: An imperative style, high-performance deep learning library. Advances in neural information processing systems. 2019;32.
51. Kingma DP, Ba J. Adam: A method for stochastic optimization. arXiv preprint arXiv:14126980. 2014.
52. Joska D, Clark L, Muramatsu N, Jericevich R, Nicolls F, Mathis A, et al. AcinoSet: a 3D pose estimation dataset and baseline models for Cheetahs in the wild. In: 2021 IEEE international conference on robotics and automation (ICRA). IEEE; 2021. p. 13901-8.
53. Zhou X, Yin T, Koltun V, Kr¨ahenbu¨hl P. Global tracking transformers. In: Proceedings of the IEEE/CVF Conference on Computer Vision and Pattern Recognition; 2022. p. 8771-80.
54. Zhang Y, Wang T, Zhang X. Motrv2: Bootstrapping end-to-end multi-object tracking by pretrained object detectors. In: Proceedings of the IEEE/CVF Conference on Computer Vision and Pattern Recognition; 2023. p. 22056-65.
55. Vidal M, Wolf N, Rosenberg B, Harris BP, Mathis A. Perspectives on individual animal identification from biology and computer vision. Integrative and comparative biology. 2021;61(3):900-16.
56. Wang Y, Xu Z, Shen H, Cheng B, Yang L. Centermask: single shot instance segmentation with point representation. In: Proceedings of the IEEE/CVF conference on computer vision and pattern recognition; 2020. p. 9313-21.
57. Liu H, Yan N, Mortazavi M, Bhanu B. Fully convolutional scene graph generation. In: Proceedings of the IEEE/CVF Conference on Computer Vision and Pattern Recognition; 2021. p. 11546-56.
58. Chong E, Wang Y, Ruiz N, Rehg JM. Detecting attended visual targets in video. In: Proceedings of the IEEE/CVF conference on computer vision and pattern recognition; 2020. p. 5396-406.
59. Woo S, Noh J, Kim K. Tackling the challenges in scene graph generation with local-to-global interactions. IEEE transactions on neural networks and learning systems. 2022;34(12):9713-26.
60. Feng S, Mostafa H, Nassar M, Majumdar S, Tripathi S. Exploiting long-term dependencies for generating dynamic scene graphs. In: Proceedings of the IEEE/CVF Winter Conference on Applications of Computer Vision; 2023. p. 5130-9.
61. Ryan F, Bati A, Lee S, Bolya D, Hoffman J, Rehg JM. Gaze-LLE: Gaze Target Estimation via Large-Scale Learned Encoders. arXiv preprint arXiv:241209586. 2024.
62. Kuhn HW. The Hungarian method for the assignment problem. Naval research logistics quarterly. 1955;2(1-2):83-97.
63. Buchanan K, Burt de Perera T, Carere C, Carter T, Hailey A, Hubrecht R, et al. Guidelines for the treatment of animals in behavioural research and teaching. 2012.

[Click here to access/download;Figure;Fig1.tiff](https://www2.cloud.editorialmanager.com/pone/download.aspx?id=41830595&guid=11c2169e-702b-43cc-af90-4a2702a18408&scheme=1)[<https://www2.cloud.editorialmanager.com/pone/download.aspx?id=41830595&guid=11c2169e-702b-43cc-af90-4a2702a18408&scheme=1>](https://www2.cloud.editorialmanager.com/pone/download.aspx?id=41830595&guid=11c2169e-702b-43cc-af90-4a2702a18408&scheme=1)

[Click here to access/download;Figure;Fig2.tiff](https://www2.cloud.editorialmanager.com/pone/download.aspx?id=41830597&guid=a57d3174-1148-43d9-ae2d-ad1126888bea&scheme=1)[<https://www2.cloud.editorialmanager.com/pone/download.aspx?id=41830597&guid=a57d3174-1148-43d9-ae2d-ad1126888bea&scheme=1>](https://www2.cloud.editorialmanager.com/pone/download.aspx?id=41830597&guid=a57d3174-1148-43d9-ae2d-ad1126888bea&scheme=1)

[Click here to access/download;Figure;Fig3.tiff](https://www2.cloud.editorialmanager.com/pone/download.aspx?id=41830598&guid=7d1d94bc-bb5e-450e-bd71-d10c9a0e7644&scheme=1)[<https://www2.cloud.editorialmanager.com/pone/download.aspx?id=41830598&guid=7d1d94bc-bb5e-450e-bd71-d10c9a0e7644&scheme=1>](https://www2.cloud.editorialmanager.com/pone/download.aspx?id=41830598&guid=7d1d94bc-bb5e-450e-bd71-d10c9a0e7644&scheme=1)

[Click here to access/download;Figure;Fig4.tiff](https://www2.cloud.editorialmanager.com/pone/download.aspx?id=41830599&guid=6f347ca3-09d6-42c8-9920-c34cb9623c00&scheme=1)[<https://www2.cloud.editorialmanager.com/pone/download.aspx?id=41830599&guid=6f347ca3-09d6-42c8-9920-c34cb9623c00&scheme=1>](https://www2.cloud.editorialmanager.com/pone/download.aspx?id=41830599&guid=6f347ca3-09d6-42c8-9920-c34cb9623c00&scheme=1)

[Click here to access/download;Figure;Fig5.tiff](https://www2.cloud.editorialmanager.com/pone/download.aspx?id=41830600&guid=b5caac31-f641-474e-9105-c5a087d9490e&scheme=1)[<https://www2.cloud.editorialmanager.com/pone/download.aspx?id=41830600&guid=b5caac31-f641-474e-9105-c5a087d9490e&scheme=1>](https://www2.cloud.editorialmanager.com/pone/download.aspx?id=41830600&guid=b5caac31-f641-474e-9105-c5a087d9490e&scheme=1)

[Click here to access/download;Figure;Fig6.tiff](https://www2.cloud.editorialmanager.com/pone/download.aspx?id=41830601&guid=6d58f2a6-83b4-4327-b1da-a5596dce2bc3&scheme=1)[<https://www2.cloud.editorialmanager.com/pone/download.aspx?id=41830601&guid=6d58f2a6-83b4-4327-b1da-a5596dce2bc3&scheme=1>](https://www2.cloud.editorialmanager.com/pone/download.aspx?id=41830601&guid=6d58f2a6-83b4-4327-b1da-a5596dce2bc3&scheme=1)

[Click here to access/download;Figure;Fig7.tiff](https://www2.cloud.editorialmanager.com/pone/download.aspx?id=41830607&guid=e582bfe4-360d-42c2-b0c3-0f56f09c517a&scheme=1)[<https://www2.cloud.editorialmanager.com/pone/download.aspx?id=41830607&guid=e582bfe4-360d-42c2-b0c3-0f56f09c517a&scheme=1>](https://www2.cloud.editorialmanager.com/pone/download.aspx?id=41830607&guid=e582bfe4-360d-42c2-b0c3-0f56f09c517a&scheme=1)

[Click here to access/download;Figure;Fig8.tiff](https://www2.cloud.editorialmanager.com/pone/download.aspx?id=41830608&guid=fd16b1a3-f82c-4525-8c56-081dc35d63eb&scheme=1)[<https://www2.cloud.editorialmanager.com/pone/download.aspx?id=41830608&guid=fd16b1a3-f82c-4525-8c56-081dc35d63eb&scheme=1>](https://www2.cloud.editorialmanager.com/pone/download.aspx?id=41830608&guid=fd16b1a3-f82c-4525-8c56-081dc35d63eb&scheme=1)
